# Drivers of the dynamics of the spread of cholera in the Democratic Republic of the Congo, 2000–2018: An eco-epidemiological study

**DOI:** 10.1371/journal.pntd.0011597

**Published:** 2023-08-28

**Authors:** Harry César Kayembe, Didier Bompangue, Catherine Linard, Bien-Aimé Mandja, Doudou Batumbo, Muriel Matunga, Jérémie Muwonga, Michel Moutschen, Hippolyte Situakibanza, Pierre Ozer

**Affiliations:** 1 Department of Basic Sciences, Faculty of Medicine, Université de Kinshasa, Kinshasa, Democratic Republic of the Congo; 2 Department of Environmental Sciences and Management, UR SPHERES, Faculty of Sciences, Université de Liège, Arlon, Belgium; 3 Chrono-Environnement, UMR CNRS 6249, Université de Franche-Comté, Besançon, France; 4 Department of Geography, Université de Namur, Namur, Belgium; 5 Graduate School Public Health Department, Adventist International Institute of Advanced Studies, Silang, Cavite, Philippines; 6 Department of Medical Biology, Faculty of Medicine, Université de Kinshasa, Kinshasa, Democratic Republic of the Congo; 7 Department of Clinical Sciences, Immunopathology—Infectious Diseases and General Internal Medicine, Université de Liège, Liege, Belgium; 8 Department of Internal Medicine, Faculty of Medicine, Université de Kinshasa, Kinshasa, Democratic Republic of the Congo; 9 Department of Parasitology and Tropical Medicine, Faculty of Medicine, Université de Kinshasa, Kinshasa, Democratic Republic of the Congo; University of Buea, CAMEROON

## Abstract

**Background:**

The dynamics of the spread of cholera epidemics in the Democratic Republic of the Congo (DRC), from east to west and within western DRC, have been extensively studied. However, the drivers of these spread processes remain unclear. We therefore sought to better understand the factors associated with these spread dynamics and their potential underlying mechanisms.

**Methods:**

In this eco-epidemiological study, we focused on the spread processes of cholera epidemics originating from the shores of Lake Kivu, involving the areas bordering Lake Kivu, the areas surrounding the lake areas, and the areas out of endemic eastern DRC (eastern and western non-endemic provinces). Over the period 2000–2018, we collected data on suspected cholera cases, and a set of several variables including types of conflicts, the number of internally displaced persons (IDPs), population density, transportation network density, and accessibility indicators. Using multivariate ordinal logistic regression models, we identified factors associated with the spread of cholera outside the endemic eastern DRC. We performed multivariate Vector Auto Regressive models to analyze potential underlying mechanisms involving the factors associated with these spread dynamics. Finally, we classified the affected health zones using hierarchical ascendant classification based on principal component analysis (PCA).

**Findings:**

The increase in the number of suspected cholera cases, the exacerbation of conflict events, and the number of IDPs in eastern endemic areas were associated with an increased risk of cholera spreading outside the endemic eastern provinces. We found that the increase in suspected cholera cases was influenced by the increase in battles at lag of 4 weeks, which were influenced by the violence against civilians with a 1-week lag. The violent conflict events influenced the increase in the number of IDPs 4 to 6 weeks later. Other influences and uni- or bidirectional causal links were observed between violent and non-violent conflicts, and between conflicts and IDPs. Hierarchical clustering on PCA identified three categories of affected health zones: densely populated urban areas with few but large and longer epidemics; moderately and accessible areas with more but small epidemics; less populated and less accessible areas with more and larger epidemics.

**Conclusion:**

Our findings argue for monitoring conflict dynamics to predict the risk of geographic expansion of cholera in the DRC. They also suggest areas where interventions should be appropriately focused to build their resilience to the disease.

## Author summary

Cholera is considered endemic in the eastern part of the Democratic Republic of the Congo (DRC), along the Great Lakes Region. From these endemic areas, cholera outbreaks regularly spread to other areas. However, some major epidemics have spread to far-off areas outside the endemic eastern DRC. Although the dynamics of their spread have been widely described, their underlying mechanisms are still unclear. Our modelling framework showed that the exacerbation of conflict events, followed by mass population movement and the amplification of cholera outbreaks in endemic areas bordering Lake Kivu, significantly increased the risk of spatio-temporal spread of major cholera epidemics outside the endemic eastern provinces of DRC. In addition, we found that the remote areas affected by these major epidemic waves were grouped into three categories based on cholera indices, population density, transportation network density, and geographic accessibility. Monitoring of conflict dynamics should be included in multisectoral cholera surveillance to anticipate the risk of significant geographic expansion of the disease in DRC.

## Introduction

Cholera is an acute diarrheal disease caused by toxigenic strains of the Gram-negative bacterium *Vibrio cholerae* [1]. To this day, the disease remains a global public health threat. Each year, it affects 1.3 to 4 million people and causes 21,000 to 143,000 deaths worldwide, predominantly in sub-Saharan Africa [2]. Since the seventh and current pandemic emerged from Indonesia in 1961, cholera has not only spread repeatedly in that part of the world since the 1970s, but has also persisted for several years [3].

In sub-Saharan Africa, cholera is heterogeneously distributed. West Africa and the Great Lakes Region (GLR) are among the regions most affected by large and recurrent epidemics [4,5]. These regions have also been or are still marked by violent wars and atrocious civil conflicts [6–8], which in turn cause complex humanitarian emergencies driven by massive population displacement, food insecurity, inadequate access to water, sanitation, and hygiene (WASH) facilities and resources, and the collapse of health systems [9–11]. In 1994, one of the worst cholera outbreaks ever occurred in the North Kivu province of the Democratic Republic of the Congo (DRC), killing nearly 50,000 refugees within the first month of the arrival of 800,000 people fleeing the Rwandan genocide [12]. Since then, cholera outbreaks have been reported annually in eastern DRC along the GLR [13]. Moreover, the lake areas of the GLR have also been identified as sources of cholera outbreaks and persistence of *Vibrio cholerae* [14].

There is substantial evidence that some major epidemics have spread from the GLR to other parts of the country, such as western DRC via the Congo River and its tributaries [3,15]. Preferential routes of these cholera spread dynamics from their epidemic foci of origin have recently been studied and identified [16]. However, despite the strongly suggested role of human population movements [17], whose flows are otherwise uninterrupted between the East and the rest of the country, the key factors and underlying mechanisms of such spread dynamics remain unclear.

Here, using data publicly available on governmental and open access digital platforms, we conducted an eco-epidemiological study to identify risk factors associated with the spatio-temporal spread of cholera epidemics outside the endemic eastern provinces of DRC. For this purpose, we focused our analysis on the routes of spread originating from the lakeshore of Lake Kivu, as these are the preferential cholera trajectories most involved in the spread of the disease out of endemic eastern DRC [16]. In addition, to better understand the potential underlying mechanisms of these cholera spread dynamics, we modelled the statistical relationships between the long-term dynamics of the different predictor variables in cholera-affected areas. Finally, we characterized the health zones outside the endemic eastern provinces that were affected by the different cholera epidemic waves. It should be noted that these health zones were affected during the east-west spread [15,17], as well as during the western secondary spread [18].

## Methods

### Data sources and collection

Data on suspected cholera cases were extracted from the DRC Integrated Disease Surveillance and Response System (IDSRS) for the period January 2000 to December 2018. In accordance with World Health Organization policy [19], any patient aged two years and older presenting with acute watery diarrhea and severe dehydration or dying from acute watery diarrhea is considered a suspected cholera cases by medical officers in each cholera treatment center and registered via line-list. The latter includes demographic, clinical, laboratory, and additional information for each case [20]. Each new outbreak is laboratory-confirmed by culture and isolation of *Vibrio cholerae* O1 from five to ten stool samples in each health zone. Subsequent cases of acute watery diarrhea in the same geographic area are assumed to be cholera [21]. Then, cholera morbidity and mortality data are aggregated electronically by Ministry of Health (MoH) officials at the health zone level. The health zone is a geographically limited area of approximately 100,000 to 300,000 inhabitants that constitutes the operational level of the structural organization of the health sector in the DRC. Each health zone is composed of one reference general hospital and 15 to 20 health centers [22]. Furthermore, the aggregated data are then transmitted to the Provincial Health Divisions, before being centralized at the Epidemiological Surveillance Directorate and reported weekly through the IDSRS (https://dhis2.fbp-rdc.org/).

We also obtained conflict data during the period 2000–2018 from a large, publicly available, high-resolution georeferenced and disaggregated conflict event dataset, the Armed Conflict Location and Event Dataset (ACLED). The latter is specifically designed to capture armed, organized political violence and demonstrations as they occur, without any restrictive threshold. Both violent and non-violent events are reported on a daily basis from cross-checking of several information sources: multiple geographic scale (local, regional, national and continental) media, reports from non-governmental or international organizations in addition to media reporting, selected social media accounts (Twitter and Telegram) and partnerships with local conflict observatories in hard-to-access cases. Types of conflict events collected are: battles, explosions/remote violence, violence against civilians, protests, riots, and strategic developments [23]. Moreover, we collected information on forced population movements during 2009–2018 (data not available prior to 2009) through daily estimates of the number of internally displaced persons (IDPs) using the Humanitarian Tools database (https://ehtools.org/) maintained by the United Nations Office for the Coordination of Humanitarian Affairs (OCHA). We then aggregated the types of conflict events and the number of IDPs on a weekly basis and at the health zone level.

Finally, we considered additional indicators associated with demographic characteristics, transportation network use, and accessibility indices. Population density data from 2000 to 2018 were extracted from annual gridded population density datasets at 1 km resolution available in the WorldPop Project’s open, high-resolution Human Distribution, Demography, and Population Dynamics geospatial databases [24]. Road and river density data were obtained from shapefiles publicly available on the open access data platform “The Humanitarian Data Exchange” managed by OCHA [25]. Using a dataset of global travel-time accessibility indicators for the year 2015, at approximately 1 km spatial resolution, travel time estimates to the nearest urban area and the nearest port were calculated in minutes, then aggregated into hours. The methodology of generating the suite of global accessibility indicators is fully described in Nelson *et al*. [26]. Our study covered a range of urban area and port sizes: (i) City between: 1,000,000–5,000,000 people; 500,000–1,000,000 people; 200,000–500,000 people; 100,000–200,000 people; (ii) Port: large size, medium size, and small size [27]. After acquisition, all these additional indicators were spatially aggregated at the health zone level (S3–S12 Figs).

### Data analysis

To identify the areas involved in the spread of epidemics out of endemic eastern DRC, we tested the spatial autocorrelation of cumulative number of suspected cholera cases over the study period in the two Kivu provinces using Moran’s I statistic based on the Queen criterion of contiguity [28]. To this end, we used local Moran’s I index as Local Indicator of Spatial Association (LISA) statistic to detect geographical areas with significant spatial correlation and clustering measures. Note that the estimated global Moran’s I statistic showed highly significant spatial autocorrelation (0.23; p< 0.008). We therefore aggregated the time series of the health zones bordering Lake Kivu (cholera endemic areas), and those of the health zones surrounding these lake areas (cholera non-endemic areas) (S1–S2 Figs).

**Fig 1:**
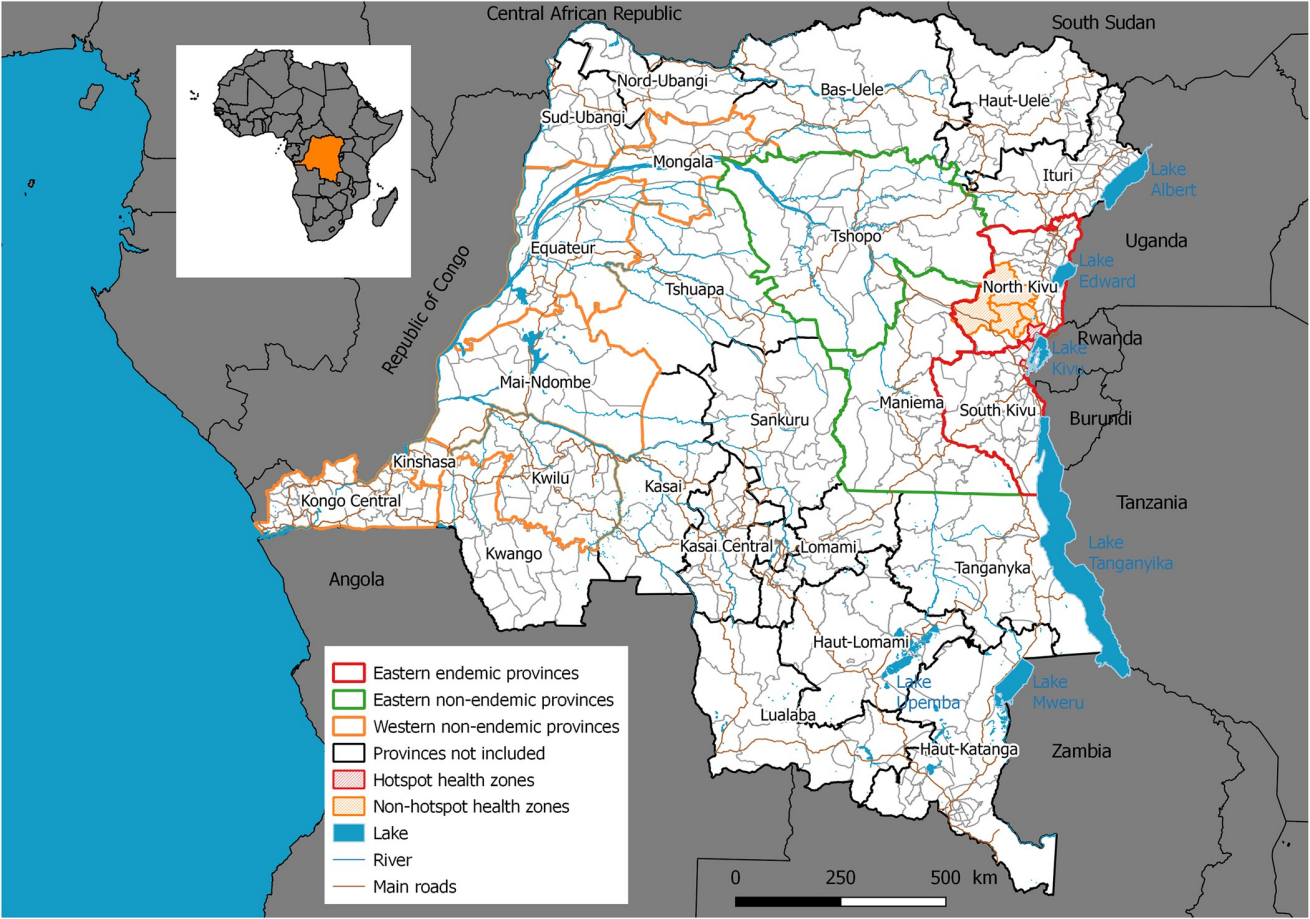
Map of the geographic areas involved in this study. Map produced in Quantum GIS version 3.8.3. using free open shapefiles of the boundaries of the health zones of the DRC from https://data.humdata.org/dataset/zones-de-sante-rdc [25].

Based on the results of Kayembe et al. study [16] and the estimations of spatial autocorrelation calculated in the previous analysis, this study focused on the following geographic areas: (i) Health zones bordering Lake Kivu as cholera endemic areas (high-high cluster in S2 Fig and hatched health zones with red borders in Fig 1); (ii) Health zones surrounding the areas bordering Lake Kivu as cholera non-endemic areas (high-low cluster in S2 Fig and hatched health zones with orange borders in Fig 1); (iii) Health zones outside the endemic eastern provinces of North and South Kivu that were affected during the east-west spread of cholera, as well as during the western secondary spread of the disease: in the eastern non-endemic provinces (with green borders in Fig 1), and the western non-endemic provinces (with orange borders in Fig 1).

We used multivariate ordinal logistic regression models to identify risk factors associated with the spatio-temporal spread of cholera epidemics outside the endemic eastern provinces of DRC, notably North and South Kivu. A regression model for ordinal responses is formally stated as follows [29]:

$$logit(P(Y\leq j))=\beta j_{0}+\beta_{1}x_{1}+\ldots +\beta_{p}x_{p},j=1,\ldots ,J-1$$ (1)

where *P*(*Y*≤*j*) is the cumulative probability *Y* of less than or equal to a specific category *j* = 1, …, *J*– 1; *β*_0_, *β*_1_,…+*β*_*p*_ are intercepts and slopes, which are different and constant for each category, respectively. In this study, the categories of the response variable correspond to the dynamics of cholera spread according to affected areas: “health zones in eastern endemic provinces (from hotspots around Lake Kivu to surrounding areas)”, “health zones in eastern non-endemic provinces”, and “health zones in western non-endemic provinces” (Fig 1). The final multivariate regression model was built with a reduced number of predictor variables selected automatically by stepwise selection methods based on minimization of the Akaike information criterion (AIC). Because the number of suspected cholera cases, types of conflict, and the number of IDPs recorded in cholera endemic and non-endemic areas followed a skewed distribution, we first log-transformed these data before performing the regression analysis.

Log-transformed data were also used to infer potential underlying mechanisms of cholera spread dynamics by modeling the statistical relationships between the long-term dynamics of predictor variables in cholera-affected areas. Here, we performed the vector autoregression (VAR) model [30]. It is a natural extension of the univariate autoregressive model to dynamic multivariate time series. VAR model also determines how each endogenous variable responds over time to a shock in its own value and in every other variable [30]. As previously suggested [31], the VAR model of order p is basically defined as:

$$y_{t}=\nu +A_{1}y_{t-1}+A_{2}y_{t-1}+\cdots +A_{t}y_{t-p}+ut$$ (2)

where *y*_*t*_ =(*y*_1*t*_, *y*_2*t*_,…,*y*_*kt*_)′ is a vector of K observable endogenous variables; *A*_*i*_ are constant coefficient matrices; *v* is a constant vector of intercept terms; *u*_t_ is a vector of white noise process. Before proceeding with the analysis of the VAR model, the stationarity of time series of log-transformed variables was tested with the Augmented Dickey Fuller (ADF) test. The optimal lag orders were chosen using the following information criteria to find the most parsimonious model [32]: AIC (n); Hannan-Quinn criterion, HQ (n); Schwarz Criterion, SC (n); Final Prediction Error criterion, FPE (n). The parameters were estimated by generalized least squares.

Although VAR coefficients capture the anticipated impact of a variable, it is generally more important to examine the model’s residuals, which represent unforeseen contemporaneous events [33]. Thus, we performed some of the common techniques used for structural analysis of VAR models. Both the Granger-causality and instantaneous causality were investigated. For both tests, the vector of endogenous variables was divided into two subvectors, *Y*_1t_ and *Y*_2t_, with dimensions *K*_1_ and *K*_2_, respectively, so that *K* = *K*_1_+*K*_2_. The subvector *Y*_1t_ was said to be Granger-causal for *Y*_2t_ if the past of *Y*_1t_ significantly helped predicting the future of *Y*_2t_ via the past of *Y*_1t_ alone [33]. Finally, we tested impulse responses based on a bootstrapped 95% confidence interval (95% CI) to describe the responses of each variable to different shocks from other predictors. It should be noted that the same methodology was recently used on the dynamics of cholera in the same geographical area [34].

Using hierarchical ascendant classification based on principal component analysis (PCA) [35], we characterized the affected health zones outside the endemic eastern provinces during the different cholera epidemic waves according to their spatial determinants: epidemiological (number of reported outbreaks, attack rate, and epidemic duration of cholera) as well as additional demographic, transport density, and accessibility indicators. Differences between groups were tested by the non-parametric Kruskal-Wallis test.

All statistical analyses were performed using the software program R version 4.1.1 and the tidyverse, spdep, rgdal, tmap, questionr, ordinal, GGally, gtsummary, tseries, vars, stargazer, and FactoMineR packages.

### Ethics approval

Ethics approval was not required because this study was carried out with routinely collected surveillance data and aggregated at the health zone level.

## Results

### Descriptive analysis results

Between 2000 and 2018, 455,333 suspected cholera cases were reported in the DRC, of which 51.2% were recorded in the two Kivu provinces, 23.3% in North Kivu, and 27.9% in South Kivu, respectively. The health zones bordering Lake Kivu accounted for 44% of all suspected cholera cases reported in the Kivu provinces. This proportion did not vary significantly over the study period (S1 Table and S13 Fig).

**Fig 2:**
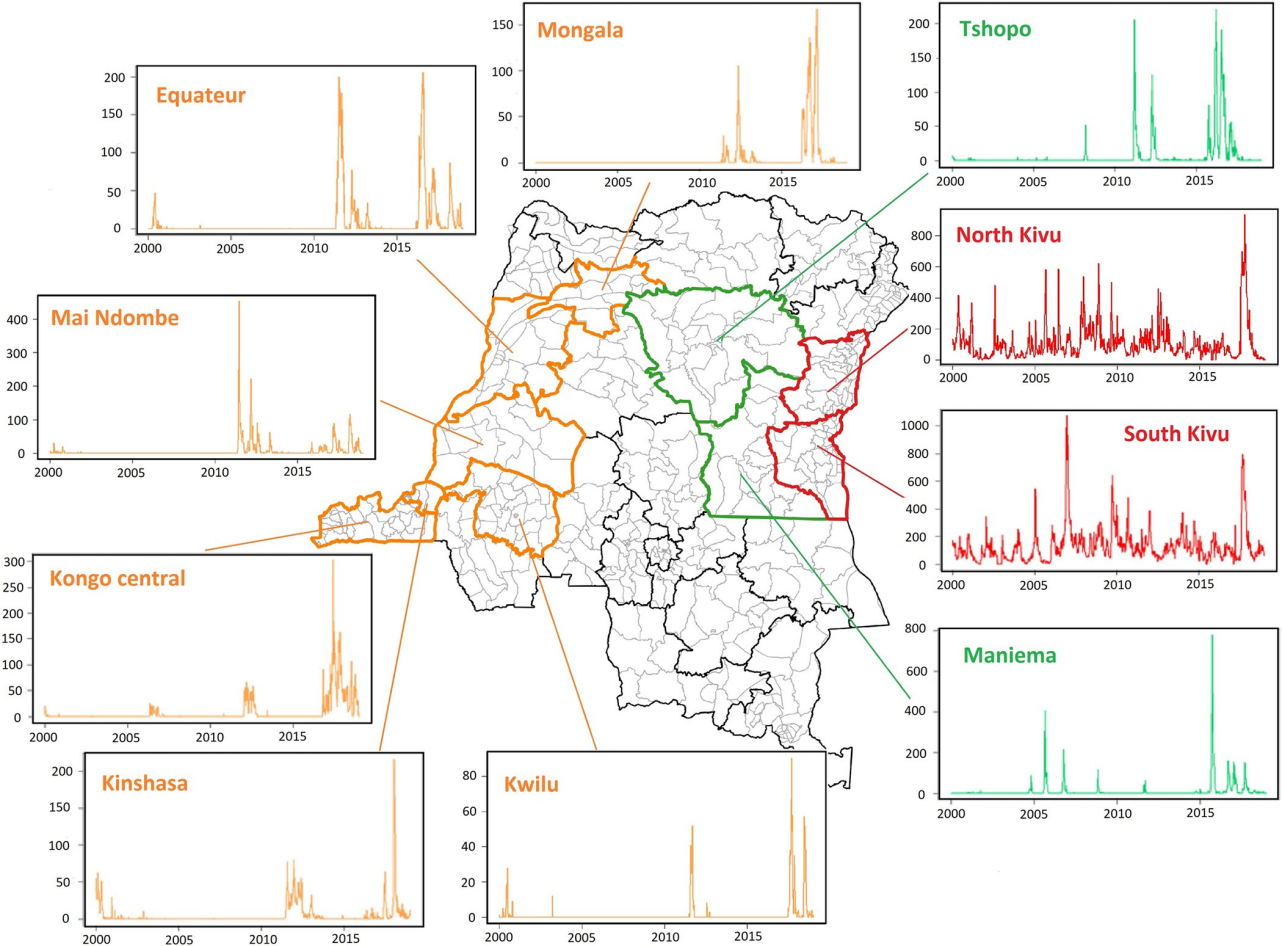
Weekly time series of suspected cholera cases reported during 2000–2018 period by provinces concerned in this study. Map produced in Quantum GIS version 3.8.3. using free open shapefiles of the boundaries of the health zones of the DRC from https://data.humdata.org/dataset/zones-de-sante-rdc [25]. Source: DRC’s IDSRS.

In the context of this study, the major epidemics that have spread outside the endemic provinces of eastern of DRC, namely North and South Kivu, affected far-off areas in: (i) eastern non-endemic provinces: Maniema (2004–2005, 2011, and 2015–2018), and Tshopo (2008, 2011–2012, and 2015–2017); (ii) western non-endemic provinces: Mongala (2011–2012, and 2016–2017), Equateur (2011–2012, and 2016–2017), Maï Ndombe (2011–2012, and 2016–2017), Kwilu (2011–2012, and 2017), Kinshasa (2011–2013, and 2016–2018), and Kongo Central (2012, and 2016–2018) (Fig 2). The other dynamics of the spread of cholera outbreaks observed in eastern and western non-endemic provinces have been described in other recent studies [16,18].

**Fig 3:**
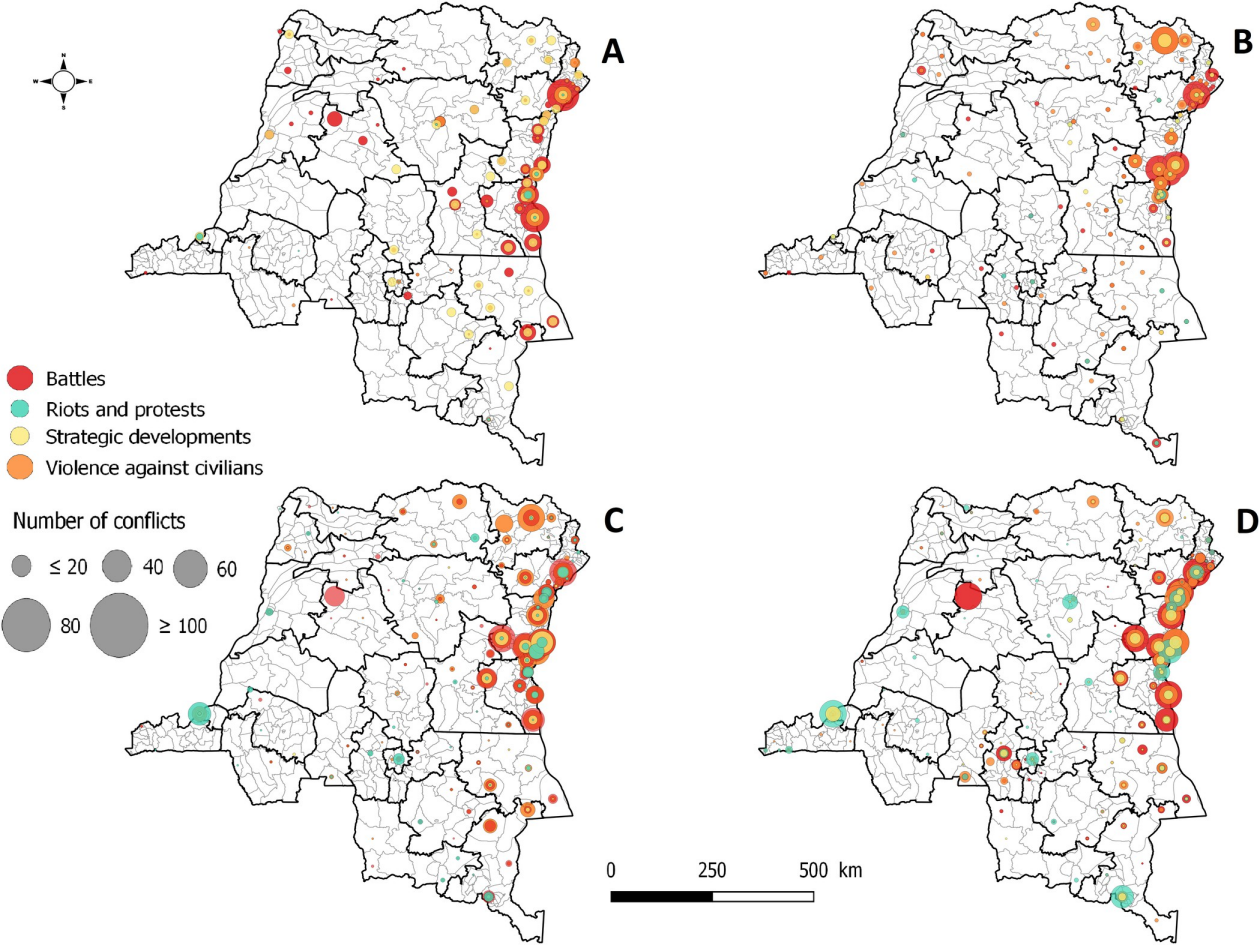
Mapping of distribution of types of conflict events in the DRC. (A) 2000–2004; (B) 2005–2009; (C) 2010–2014; (D) 2015–2018. Remote violence events were excluded because they were underrepresented during the study period. Maps produced in Quantum GIS version 3.8.3. using free open shapefiles of the boundaries of the health zones of the DRC from https://data.humdata.org/dataset/zones-de-sante-rdc [25]. Source: ACLED.

During the study period, 11,980 conflict events were reported in the DRC. Of these conflict events, battles were in majority (44.5%), followed by violence against civilians (34.0%), riots and protests (11.9%), strategic developments (8.6%), and remote violence (0.9%) (S2 Table). With the exception of riots and protests, more than half of the recorded types of conflicts were concentrated in the two Kivu provinces, with twice as many in North Kivu: battles (53.6%), strategic developments (57.8%), and violence against civilians (52.1%). Beyond the three-fold increase in the number of reported conflicts in North and South Kivu during the second decade (S14 Fig), the cholera endemic areas around Lake Kivu accounted for half of the riots and protests, and one third of the other violent and non-violent conflicts (Fig 3, and S3–S5 Tables).

**Fig 4:**
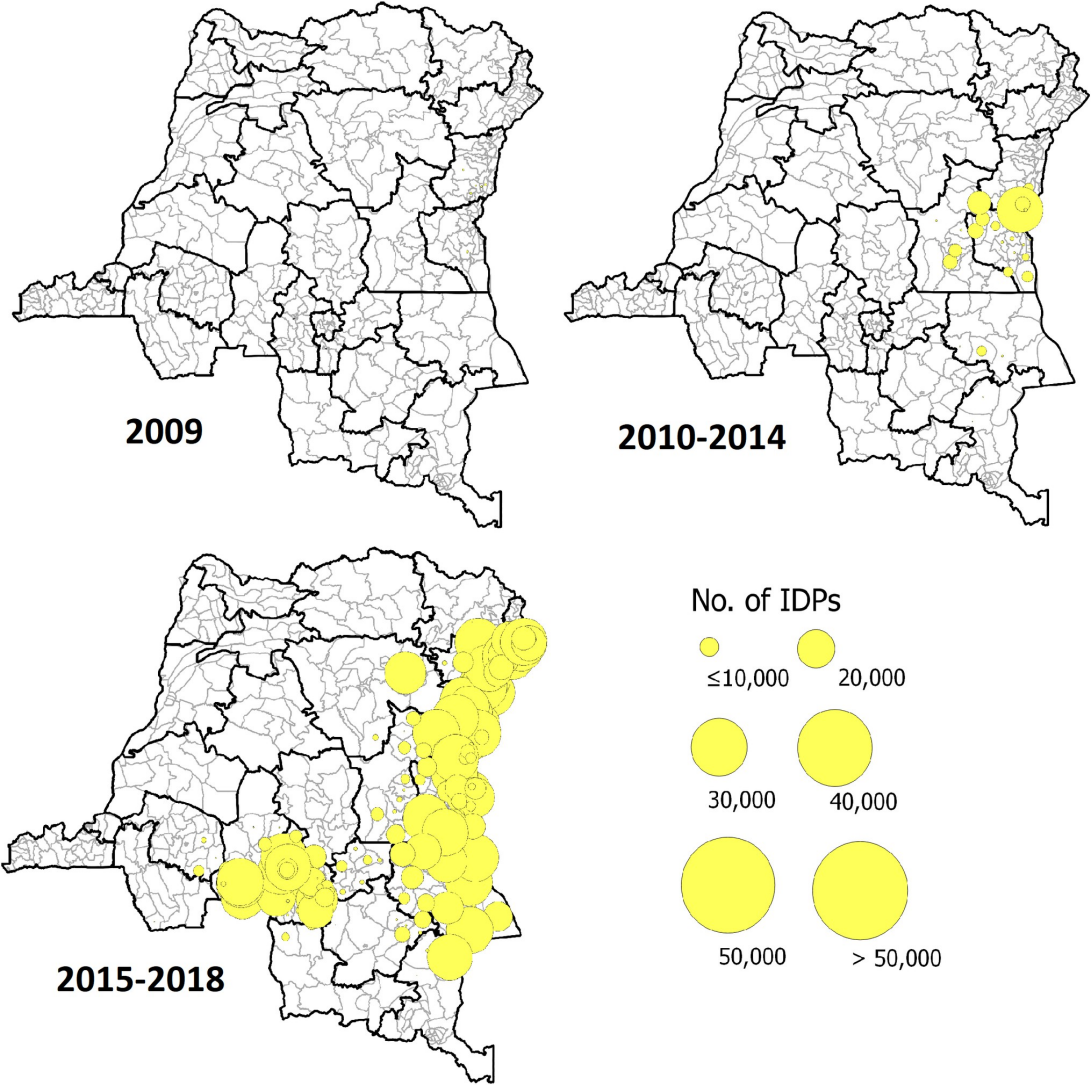
Mapping of distribution of the number of IDPs in the DRC, 2009–2018. Maps produced in Quantum GIS version 3.8.3. using free open shapefiles of the boundaries of the health zones of the DRC from https://data.humdata.org/dataset/zones-de-sante-rdc [25]. Source: Humanitarian Tools database.

Concerning forced migration, from 2009 to 2018, more than 5.7 million IDPs were reported in the DRC, with a substantial increase since 2015 (from nearly 320,000 to more than 5.4 million). North and South Kivu provinces accounted for 45% of forced displacement nationwide. Although 3% of these IDPs were recorded in the Lake Kivu endemic areas (compared to 20% in the surrounding areas), the heavily cholera-affected health zones around these lake areas had 75% more IDPs than the less or unaffected health zones (S6–S8 Tables and Fig 4).

### Risk factors associated with the spatio-temporal spread of cholera epidemics outside the endemic eastern provinces of DRC

**Fig 5:**
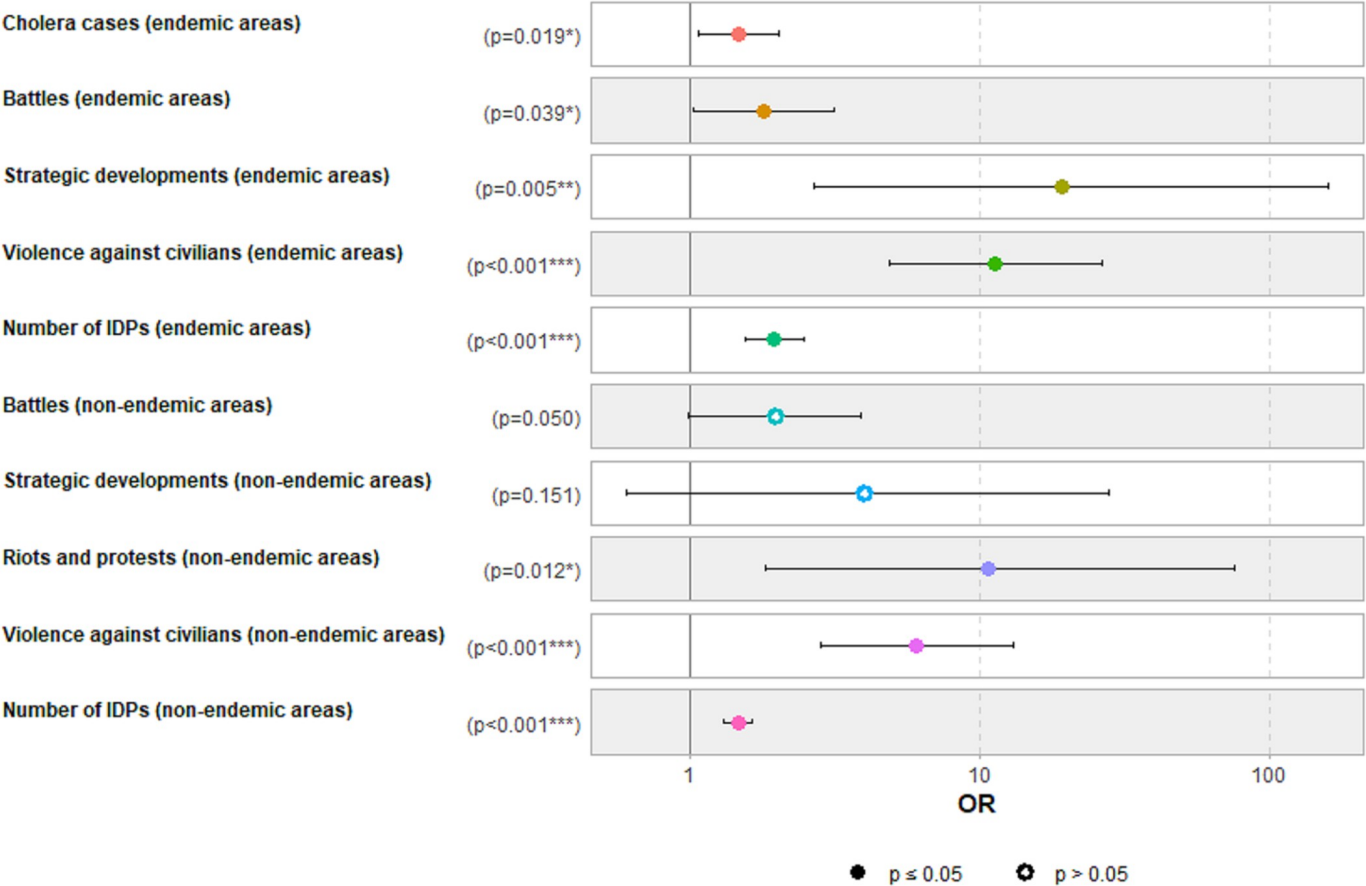
Factors associated with the spatio-temporal spread of cholera epidemics outside the endemic eastern provinces (North and South Kivu). Sources: DRC’s IDSRS, ACLED, and Humanitarian Tools database.

In the final multivariate ordinal logistic regression model (Fig 5), the spatio-temporal spread of cholera epidemics outside the endemic eastern provinces of DRC (North and South Kivu) was associated with exacerbation of the number of suspected cholera cases (Odds ratio [OR] = 1.47; 95% confidence interval [95% CI] = 1.07–2.04), battles (OR = 1.81; 95% CI = 1.03–3.17), the number of IDPs (OR = 1.94; 95% CI = 1.55–2.49), violence against civilians (OR = 11.2; 95% CI = 4.86–26.5), and strategic developments (OR = 19.2; 95% CI = 2.67–160) in cholera endemic areas. On the other hand, it was associated with exacerbation of the number of IDPs (OR = 1.47; 95% CI = 1.31–1.65), violence against civilians (OR = 6.04; 95% CI = 2.83–13.1), and riots and protests (OR = 10.8; 95% CI = 1.82–75.9) in cholera non-endemic areas. Overall, the final regression model explained about 33% of all variance in the risk of the spread of cholera outside the endemic eastern DRC (Nagelkerke R2 = 0.33).

### Potential underlying mechanisms involving factors associated with the spread of cholera outside the endemic eastern DRC

Analysis of linear interdependencies between time series of variables using multivariate VAR models showed the following (Table 1): In cholera endemic areas: (i) battles have influenced the number of suspected cholera cases, strategic developments, and violence against civilians; (ii) violence against civilians has influenced strategic developments, and the number of IDPs; (iii) riots and protests were influenced by the number of IDPs; (iv) strategic developments, and the number of IDPs have influenced violence against civilians. In cholera non-endemic areas: (i) both violence against civilians and the number of IDPs have influenced battles, strategic developments, and riots and protests; (ii) violence against civilians was influenced by strategic developments, riots and protests, and the number of IDPs; (iii) the number of IDPs was influenced by battles, and riots and protests.

**Table 1:** Linear interdependencies between time series of the six variables considered using multivariate VAR models.

	Cholera endemic areas				Cholera non-endemic areas			
Independent variables	Number of cholera cases (dependent variable)				Number of cholera cases (dependent variable)			
	Estimate	Std. Error	t-value	Pr (>|t|)	Estimate	Std. Error	t-value	Pr (>|t|)
Battles	0.097	0.029	3.329	**0.001**	0.012	0.059	0.201	0.841
Strategic developments	0.009	0.078	0.111	0.911	-0.236	0.160	-1.474	0.141
Riots and protests	0.087	0.080	1.083	0.279	-0.128	0.147	-0.868	0.385
Violence against civilians	-0.015	0.039	-0.389	0.698	-0.023	0.066	-0.345	0.730
Number of IDPs	-0.004	0.008	-0.457	0.648	-0.014	0.010	-1.362	0.173
Constant	0.232	0.030	4.187	7.689	0.201	0.035	5.653	<0.001
	Battles (dependent variable)				Battles (dependent variable)			
	Estimate	Std. Error	t-value	Pr (>|t|)	Estimate	Std. Error	t-value	Pr (>|t|)
Number of cholera cases	0.007	0.016	0.421	0.674	0.027	0.018	1.498	0.135
Strategic developments	0.042	0.080	0.523	0.601	0.128	0.087	1.460	0.145
Riots and protests	-0.080	0.083	-0.966	0.334	-0.021	0.079	-0.258	0.796
Violence against civilians	0.060	0.040	1.493	0.136	0.099	0.036	2.799	**0.005**
Number of IDPs	-0.007	0.008	-0.791	0.429	0.014	0.005	2.594	**0.010**
Constant	0.056	0.031	1.797	0.073	0.071	0.017	4.118	<0.001
	Strategic developments (dependent variable)				Strategic developments (dependent variable)			
	Estimate	Std. Error	t-value	Pr (>|t|)	Estimate	Std. Error	t-value	Pr (>|t|)
Number of cholera cases	-0.004	0.006	-0.603	0.547	0.001	0.004	0.193	0.847
Battles	0.064	0.012	5.246	**<0.001**	0.015	0.012	1.307	0.192
Riots and protests	0.007	0.034	0.210	0.834	0.051	0.029	1.747	0.081
Violence against civilians	0.072	0.016	4.368	**<0.001**	0.029	0.012	2.317	**0.021**
Number of IDPs	0.002	0.003	0.653	0.514	0.004	0.002	2.026	**0.043**
Constant	0.010	0.013	0.767	0.443	0.004	0.006	0.617	0.537
	Riots and protests (dependent variable)				Riots and protests (dependent variable)			
	Estimate	Std. Error	t-value	Pr (>|t|)	Estimate	Std. Error	t-value	Pr (>|t|)
Number of cholera cases	-0.007	0.006	-1.065	0.287	-0.000	0.005	-0.026	0.979
Battles	0.002	0.012	0.191	0.849	0.003	0.014	0.238	0.812
Strategic developments	0.011	0.031	0.361	0.718	0.021	0.037	0.555	0.579
Violence against civilians	0.005	0.016	0.298	0.766	0.033	0.014	2.310	**0.021**
Number of IDPs	0.007	0.003	2.238	**0.025**	0.005	0.002	2.379	**0.018**
Constant	0.028	0.012	2.271	0.023	0.008	0.007	1.173	0.241
	Violence against civilians (dependent variable)				Violence against civilians (dependent variable)			
	Estimate	Std. Error	t-value	Pr (>|t|)	Estimate	Std. Error	t-value	Pr (>|t|)
Number of cholera cases	-0.019	0.012	-1.532	0.126	-0.012	0.010	-1.150	0.250
Battles	0.105	0.023	4.521	**<0.001**	0.023	0.029	0.795	0.427
Strategic developments	0.318	0.061	5.182	**<0.001**	0.194	0.081	2.397	**0.017**
Riots and protests	0.086	0.064	1.350	0.177	0.165	0.073	2.271	**0.023**
Number of IDPs	0.027	0.006	4.184	**<0.001**	0.014	0.005	3.085	**0.002**
Constant	0.067	0.024	2.798	0.005	0.061	0.015	4.005	<0.001
	Number of IDPs (dependent variable)				Number of IDPs (dependent variable)			
	Estimate	Std. Error	t-value	Pr (>|t|)	Estimate	Std. Error	t-value	Pr (>|t|)
Number of cholera cases	-0.105	0.060	-1.751	0.080	0.099	0.066	1.496	0.135
Battles	0.017	0.111	0.153	0.878	0.754	0.189	3.981	**<0.001**
Strategic developments	0.099	0.296	0.334	0.738	0.381	0.520	0.733	0.464
Riots and protests	0.682	0.307	2.221	**0.027**	1.718	0.469	3.663	**<0.001**
Violence against civilians	0.449	0.150	2.987	**0.003**	0.266	0.198	1.341	0.180
Constant	0.314	0.115	2.722	0.007	0.119	0.097	1.224	0.221

Abbreviations: IDPs, internally displaced persons; Std. Error, Standard Error; Pr(>|t|), p-value associated with the t-value. Sources: DRC’s IDSRS, ACLED, and Humanitarian Tools database.

The results of the Granger causality and instantaneous causality tests for the six variables included in the analysis were summarized as follows (Table 2): In cholera endemic areas: (i) battles Granger caused suspected cholera cases, strategic developments, and the number of IDPs; (ii) battles and violence against civilians as well as strategic developments and violence against civilians were mutually instantaneous and Granger caused; (iii) the number of IDPs and violence against civilians as well as the number of IDPs and riots and protests were mutually Granger caused. In cholera non-endemic areas: (i) battles and strategic developments as well as battles and the number of IDPs, and the number of IDPs and riots and protests were mutually instantaneous and Granger caused; (ii) violence against civilians and riots and protests were mutually Granger caused; (iii) the number of IDPs instantaneously and Granger caused violence against civilians, and the latter caused strategic developments.

**Table 2:** Instantaneous and Granger causality tests for the six variables considered.

	Cholera endemic areas				Cholera non-endemic areas			
Cause variable	Number of cholera cases (effect variable)				Number of cholera cases (effect variable)			
	Granger causality tests		Instantaneous causality tests		Granger causality tests		Instantaneous causality tests	
	F-Test	p-value	Chi-squared	p-value	F-Test	p-value	Chi-squared	p-value
Battles	6.461	**0.002**	0.178	0.673	0.145	0.933	0.001	0.973
Strategic developments	0.793	0.623	0.671	0.736	0.806	0.447	1.781	0.182
Riots and protests	0.968	0.424	0.302	0.583	1.856	0.073	0.477	0.490
Violence against civilians	1.196	0.311	1.447	0.229	1.751	0.106	1.053	0.305
Number of IDPs	0.721	0.691	2.954	0.086	1.946	0.050	2.652	0.103
	Battles (effect variable)				Battles (effect variable)			
	Granger causality tests		Instantaneous causality tests		Granger causality tests		Instantaneous causality tests	
	F-Test	p-value	Chi-squared	p-value	F-Test	p-value	Chi-squared	p-value
Number of cholera cases	1.134	0.339	0.074	0.785	2.109	0.097	0.001	0.973
Strategic developments	1.182	0.315	6.711	**0.010**	4.413	**<0.001**	19.373	**<0.001**
Riots and protests	0.943	0.419	0.007	0.934	3.938	**0.008**	0.063	0.802
Violence against civilians	2.654	**0.007**	23.707	**<0.001**	2.824	**0.003**	30.804	**<0.001**
Number of IDPs	1.678	0.089	4.003	**0.045**	3.115	**0.002**	7.606	**0.006**
	Strategic developments (effect variable)				Strategic developments (effect variable)			
	Granger causality tests		Instantaneous causality tests		Granger causality tests		Instantaneous causality tests	
	F-Test	p-value	Chi-squared	p-value	F-Test	p-value	Chi-squared	p-value
Number of cholera cases	0.671	0.736	1.148	0.284	0.708	0.685	1.661	0.198
Battles	4.731	**<0.001**	6.250	**0.012**	3.417	**0.002**	19.373	**<0.001**
Riots and protests	1.246	0.262	10.9	**<0.001**	1.719	0.080	1.549	0.213
Violence against civilians	3.119	**<0.001**	50.812	**<0.001**	2.956	**0.002**	10.125	**0.001**
Number of IDPs	1.635	0.100	3.937	**0.047**	2.297	**0.019**	1.512	0.219
	Riots and protests (effect variable)				Riots and protests (effect variable)			
	Granger causality tests		Instantaneous causality tests		Granger causality tests		Instantaneous causality tests	
	F-Test	p-value	Chi-squared	p-value	F-Test	p-value	Chi-squared	p-value
Number of cholera cases	0.407	0.804	0.302	0.583	0.615	0.744	0.477	0.490
Battles	0.228	0.877	0.007	0.934	0.802	0.492	0.063	0.802
Strategic developments	1.540	0.128	10.9	**<0.001**	4.155	**<0.001**	1.549	0.213
Violence against civilians	0.792	0.610	1.162	0.281	2.941	**0.002**	1.323	0.250
Number of IDPs	2.092	**0.027**	0.182	0.670	4.290	**<0.001**	7.687	**0.006**
	Violence against civilians (effect variable)				Violence against civilians (effect variable)			
	Granger causality tests		Instantaneous causality tests		Granger causality tests		Instantaneous causality tests	
	F-Test	p-value	Chi-squared	p-value	F-Test	p-value	Chi-squared	p-value
Number of cholera cases	2.336	0.053	1.447	0.229	0.895	0.498	1.053	0.305
Battles	4.136	**<0.001**	23.707	**<0.001**	1.065	0.381	31.533	**<0.001**
Strategic developments	5.791	**<0.001**	50.812	**<0.001**	0.762	0.652	10.125	**0.001**
Riots and protests	0.792	0.610	1.162	0.281	2.941	**0.002**	1.323	0.250
Number of IDPs	3.798	**0.002**	1.278	0.258	3.393	**0.009**	4.565	**0.033**
	Number of IDPs (effect variable)				Number of IDPs (effect variable)			
	Granger causality tests		Instantaneous causality tests		Granger causality tests		Instantaneous causality tests	
	F-Test	p-value	Chi-squared	p-value	F-Test	p-value	Chi-squared	p-value
Number of cholera cases	1.103	0.357	2.954	0.086	1.071	0.360	3.196	0.074
Battles	2.088	**0.028**	4.003	**0.045**	5.137	**0.001**	8.177	**0.004**
Strategic developments	1.318	0.222	3.937	**0.047**	1.063	0.364	2.129	0.145
Riots and protests	2.697	**0.004**	0.182	0.670	5.257	**<0.001**	7.126	**0.008**
Violence against civilians	4.895	**<0.001**	1.278	0.258	1.262	0.283	4.565	**0.033**

Abbreviation: IDPs: internally displaced persons. Sources: DRC’s IDSRS, ACLED, and Humanitarian Tools database.

Impulse response tests revealed that (S15–S37 Figs): In cholera endemic areas: (i) battles led to an increase in suspected cholera cases, with the most significant impact occurring at lag of 4 weeks, as well as strategic developments (2 weeks), violence against civilians (2 weeks), and the number of IDPs (6 weeks); (ii) violence against civilians resulted in the largest increase in battles, strategic developments, and the number of IDPs at lags of 1 week, 1 week, and 4 weeks, respectively; (iii) the highest positive effect of the number of IDPs on riots and protests, and violence against civilians was observed at lags of 6 weeks and 2 weeks, respectively. In cholera non-endemic areas: (i) battles and violence against civilians, as well as battles and the number of IDPs, and the number of IDPs and riots and protests mutually increased significantly at lags of 1 week, 4 weeks and 8 weeks, respectively; (ii) violence against civilians led to the largest increase in the number of IDPs, and riots and protests at lags of 1 week and 7 weeks respectively (4 and 8 weeks vice versa).

### Characterization of the affected health zones outside the endemic eastern provinces

**Fig 6:**
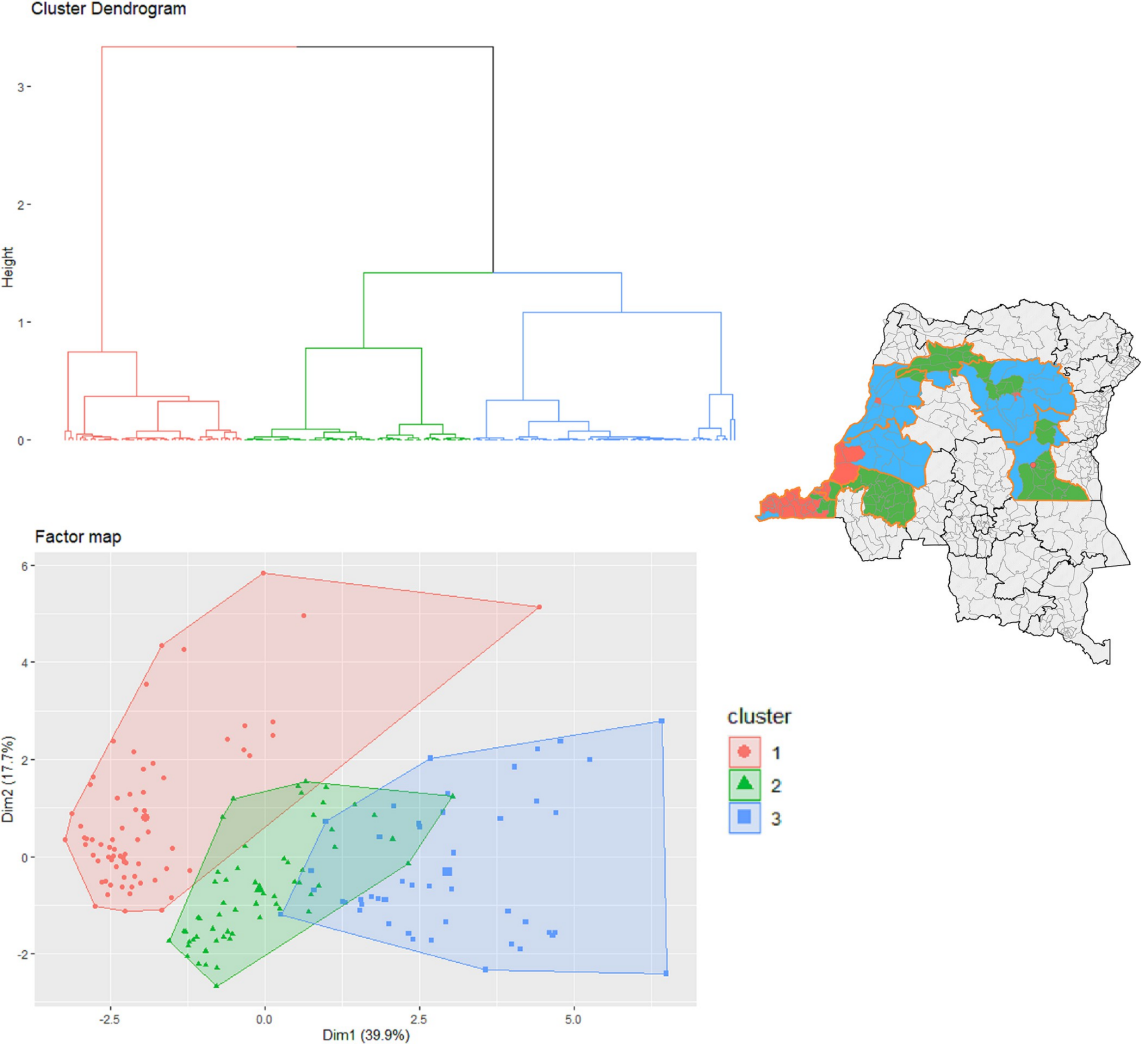
Classification and mapping of the affected health zones outside the endemic eastern DRC regarding spatial determinants based hierarchical clustering on PCA. Map produced in Quantum GIS version 3.8.3. using free open shapefiles of the boundaries of the health zones of the DRC from https://data.humdata.org/dataset/zones-de-sante-rdc [25]. Sources: DRC’s IDSRS, WorldPop, Humanitarian Data Exchange, and Global travel-time accessibility indicators.

Hierarchical ascendant classification based on PCA classified the affected health zones outside the endemic eastern provinces into three categories represented as clusters. They were mapped and detailed in Fig 6 and Table 3, respectively. Cluster 1 was associated with both low number of outbreaks but high attack rate of cholera, longer epidemic duration (median: 25 weeks), high population density (median: 634 people/km^2^), and relatively short average travel time to both the nearest city (for different sets of urban areas) and the nearest port (for different sizes). Cluster 2 was associated with both high number of outbreaks but low attack rate of cholera, short epidemic duration (median: 9 weeks), medium population density (median: 39 people/km^2^), higher river density (median: 0.14), and high average travel time to both the nearest city (for different sets of urban areas) and the nearest port (for different sizes). Cluster 3 was associated with both high number of outbreaks and high attack rate of cholera, long epidemic duration (median: 16 weeks), low population density (median: 12 people/km^2^), high river density (median: 0.12), and higher average travel time to both the nearest city (for different sets of urban areas) and the nearest port (for different sizes).

**Table 3:** Detailed classification of the health zones affected during the spread of cholera epidemics according to spatial determinants.

Characteristics	Cluster 1 (n = 69)	Cluster 2 (n = 59)	Cluster 3 (n = 48)
Number of reported cholera outbreaks	1 (1–3)	2 (2–2)	2 (1–4)
Cholera attack rates (per 100,000 inhabitants)	2.7 (0.6–17.1)	2.3 (0–10)	5.5 (0.9–20.3)
Cholera epidemic duration (weeks)	25 (11–51)	9 (0–39)	16 (7–51)
Population density	634 (78–20,059)	39 (23–61)	12 (7–19)
River density	0.09 (0.08–0.10)	0.14 (0.12–0.16)	0.12 (0.10–0.14)
Road density	0.05 (0.04–0.06)	0.08 (0.06–0.11)	0.05 (0.04–0.06)
Travel time to the nearest city between 1,000,000–5,000,000 people (hours)	16 (14–19)	19 (19–23)	26 (23–32)
Travel time to the nearest city between 500,000–1,000,000 people (hours)	7 (7–7)	12 (10–14)	24 (17–31)
Travel time to the nearest city between 200,000–500,000 people (hours)	4 (4–5)	8 (4–14)	17 (12–20)
Travel time to the nearest city between 100,000–200,000 people (hours)	3 (3–4)	7 (6–9)	17 (12–21)
Travel time to the nearest large port (hours)	45 (45–45)	49 (46–50)	57 (55–61)
Travel time to the nearest medium port (hours)	26 (26–27)	32 (31–34)	42 (38–47)
Travel time to the nearest small port (hours)	3 (3–4)	29 (11–34)	37 (32–42)

Results are expressed as median (interquartile range). Sources: DRC’s IDSRS, WorldPop, Humanitarian Data Exchange, and Global travel-time accessibility indicators.

The subsequent Kruskal-Wallis tests showed that all differences between the three groups based on each characteristic were statistically significant (S38 Fig).

## Discussion

Our findings suggest that the exacerbation of violent and non-violent conflict events and the number of IDPs, as well as the increase in the number of cholera cases in the endemic eastern provinces of North and South Kivu were associated with a heightened risk of geographic country widespread of cholera outbreaks. Such a trend in cholera cases, especially in hotspot areas along the GLR, reinforces the hypothesis of incubator functioning in the expansion of the disease across the country [15]. Moreover, similar dynamics have been observed elsewhere, including in Yemen, where the civil war has led to a massive humanitarian crisis, creating conditions conducive to the largest and fastest spreading cholera epidemic worldwide [36]. Although a little is known about the actual impact of conflict on the spread of infectious diseases, these observations shed additional insight into their complex but indisputable link.

The results of this study highlighted potential underlying mechanisms involving factors associated with the spread of cholera outside the endemic eastern DRC. We found a variety of influences and causal links between conflicts and cholera cases, between types of conflicts, and between conflicts and IDPs. In cholera endemic areas, the exacerbation of battles has significantly influenced the increase in suspected cholera cases 4 weeks later. This trend in cholera cases would also be due to the mediating effect of the battles, which were influenced by the violence against civilians a week earlier. These violent conflict events also contributed to the increase in the number of IDPs and strategic developments across two lag-ranges, 4 to 6 weeks and 1 to 2 weeks, respectively. On the other hand, in cholera non-endemic areas, we observed mutual influences and bidirectional causalities between violent and non-violent conflicts, as well as between conflicts events and internal displacement. However, there was a unidirectional dominance from violent conflicts to the dynamics of IDPs (1 week later), as well as to non-violent events (7 weeks later).

As demonstrated above, the greater the number of reported conflict events in cholera endemic and non-endemic areas, the greater the risk of geographic spread of the disease. Specifically, the significant intensification of conflict events and the subsequent worsening in the movement of IDPs and cholera outbreaks in endemic areas would have played an amplifying role in high-risk channels for the spread of major epidemics outside the endemic eastern provinces. In a conflict-fueled humanitarian crisis, the risk of clean water shortages, and poor sanitation and hygiene practices in IDP camps and host communities is increased tenfold [10,11]. Also, in conflict-affected areas, it is expected that some of the fleeing IDPs may have already contracted a cholera infection, either asymptomatic or incubating, or may be hosted in areas where a cholera outbreak has already occurred [37]. All this is in addition to the regular spread of cholera along the lake areas and to surrounding health zones, due to multifactorial vulnerabilities, such as structural population mobility, water supply interruptions, and high population density [14,38].

After classifying the health zones located outside the endemic eastern provinces that were affected during the spread of cholera epidemics, we found that health zones with high accessibility reported few epidemics, but these were large and of longer duration, and were densely populated. A second category of health zones reporting more cholera outbreaks, but of smaller magnitude and shorter duration, was moderately populated and accessible. The last category was characterized by greater number of reported cholera outbreaks, of greater magnitude and long duration, but sparsely populated and inaccessible areas. This finding is consistent with other studies that have shown that attack rates and duration of cholera epidemics are higher and longer, respectively, in sub-Saharan urban areas [39]. The latter are at increased risk of communicable diseases, including diarrheal diseases such as cholera, due to the existence of informal settlements associated with rapid urbanization and overcrowding, with inadequate sanitation facilities and basic services [40]. However, population density is not always related to severe cholera outbreak outcomes [39]. In our context, this is illustrated by the apparently controversial estimates observed in category 3 health zones. This may be because the delays between cholera alerts and response would be much longer in hard-to-reach rural areas than in urban areas [41].

Our study had some limitations that were clearly identified and partially resolved. We only used data on suspected cholera cases. Given the low sample testing rates for each new outbreak due to limited resources [21], the use of suspected cases could lead to an overestimation or underestimation of the disease burden. Furthermore, it has already been shown that a fraction of suspected cases are likely to be true cholera cases [42–44]. Biological confirmation and genomic data would have allowed accurate estimation of cholera incidence and transmission dynamics of circulating *Vibrio cholerae* strains, respectively. Nevertheless, the assessment of the adequacy of surveillance and response to epidemic-prone diseases reported weekly and monitored by the DRC’s IDSRS showed the highest level for cholera, demonstrating the relevance of data on suspected cases for epidemiological research or public health purposes [45]. In addition, another assessment of IDSR key performance indicators previously indicated that the DRC is among the African countries with high coverage of IDSR implementation at the subnational level in terms of training, timeliness and completeness of reporting [46].

Another limitation may be related to the difficulty of obtaining certain information in developing countries, particularly in hard-to-reach areas. Conflict data is drawn from multiple information sources, including online data and self-reported data from local sources. This can lead to inherent biases, especially in areas where the level of cell phone and internet penetration is largely insufficient. However, ACLED data are not limited by arbitrary thresholds that could mask low-intensity violent events, violence related to unidentified or anonymous armed agents and other perpetrators, as well as other forms of public disorder [47]. In addition, several previous works have shown that ACLED probably represents the most reliable dataset for point data analyses at different geographic scales (continental, national, and sub-national) based on coverage levels, depth, ease of use, and content [48–51]. Thus, the likelihood of underreporting of conflict events in the ACLED is lower than in other public, open-source conflict datasets with global coverage. Furthermore, data on conflict-induced displacement are based on the number of IDPs reported in a given health zone. We were unable to acquire sufficient data on the movement of IDPs between health zones due to limitations in mobility tracking data, which were either incomplete between 2009 and 2018 (https://ehtools.org/) or not available before 2016 (https://dtm.iom.int/democratic-republic-congo). Despite this limitation, trends in the number of IDPs and other predictor variables involved in the spread dynamics studied were generally consistent. Additional spatial determinants extracted from multiple open access sources were considered, ignoring possible variations that may have existed over the study period. Migration flows in a geographic area may be influenced by seasonal cycles due to the navigability or practicability of transportation networks.

Finally, we were not able to integrate the socioeconomic variables such as WASH indicators. It was not possible to collect them annually, let alone weekly, at the health zone level, except during major surveys such as the Demographic and Health Survey [52] or Multiple Indicator Cluster Survey [53], which are routinely conducted at the provincial level every seven years in the DRC. Nevertheless, socioeconomic conditions are almost similar in the cholera-endemic eastern provinces considered in this study, in terms of proportion of households using clean water, improved hygiene and sanitation facilities (S39–S42 Figs). In addition, the epidemiological, conflict, and population displacement covariates included in the final ordinal logistic regression model were quite highly predictive of the spatio-temporal spread of cholera epidemics outside the endemic eastern regions of the DRC, with a pseudo-R2 value of 0.33. As noted elsewhere [54], it can be concluded that the absence of socioeconomic variables in the model is less likely to call into question the validity and consistency of the results obtained. Furthermore, more large-scale surveys are needed to make WASH indicators available systematically and at a finer geographic scale.

## Conclusions

In conclusion, our findings highlight the impact of conflict on forced migration and the dynamics of cholera outbreaks in endemic areas bordering Lake Kivu, as well as the subsequent spread of major cholera epidemics to other areas in the non-endemic eastern and western provinces. The level of vulnerability of these areas affected by diffusion processes [16,18] have also been described using different risk typologies. The inclusion of conflict dynamics monitoring in the early warning, alert and response system of integrated cholera surveillance can help anticipate the risk of significant expansion of epidemics in the DRC. In addition, consideration of risk typologies in the implementation of prevention or response strategies can help improve efforts in the fight against cholera under a context of limited resources.

## Supporting information

S1 Fig Moran’s I statistics of spatial autocorrelation of cholera cases reported at the health zone level, North and South Kivu provinces, 2000–2018. Source: DRC’s IDSRS.
(TIF)

S2 Fig LISA spatial clustering pattern of reported cholera cases at the health zone level, North and South Kivu provinces, 2000–2018. Source: DRC’s IDSRS.
(TIF)

S3 Fig Population density, 2000–2018. The green borders correspond to provinces involved in our study. Map produced in Quantum GIS version 3.8.3. using free open shapefiles of the boundaries of the health zones of the DRC from https://data.humdata.org/dataset/zones-de-sante-rdc [25]. Source: Worldpop.
(TIF)

S4 Fig Road density, 2000–2018. The green borders correspond to provinces involved in our study. Map produced in Quantum GIS version 3.8.3. using free open shapefiles of the boundaries of the health zones of the DRC from https://data.humdata.org/dataset/zones-de-sante-rdc [25]. Source: Humanitarian Data Exchange.
(TIF)

S5 Fig River density, 2000–2018. The green borders correspond to provinces involved in our study. Map produced in Quantum GIS version 3.8.3. using free open shapefiles of the boundaries of the health zones of the DRC from https://data.humdata.org/dataset/zones-de-sante-rdc [25]. Source: Humanitarian Data Exchange.
(TIF)

S6 Fig Travel time to the nearest urban area between 1,000,000–5,000,000 people. The green borders correspond to provinces involved in our study. Map produced in Quantum GIS version 3.8.3. using free open shapefiles of the boundaries of the health zones of the DRC from https://data.humdata.org/dataset/zones-de-sante-rdc [25]. Source: Global travel-time accessibility indicators.
(TIF)

S7 Fig Travel time to the nearest urban area between 500,000–1,000,000 people. The green borders correspond to provinces involved in our study. Map produced in Quantum GIS version 3.8.3. using free open shapefiles of the boundaries of the health zones of the DRC from https://data.humdata.org/dataset/zones-de-sante-rdc [25]. Source: Global travel-time accessibility indicators.
(TIF)

S8 Fig Travel time to the nearest urban area between 200,000–500,000 people. The green borders correspond to provinces involved in our study. Map produced in Quantum GIS version 3.8.3. using free open shapefiles of the boundaries of the health zones of the DRC from https://data.humdata.org/dataset/zones-de-sante-rdc [25]. Source: Global travel-time accessibility indicators.
(TIF)

S9 Fig Travel time to the nearest urban area between 100,000–200,000 people. The green borders correspond to provinces involved in our study. Map produced in Quantum GIS version 3.8.3. using free open shapefiles of the boundaries of the health zones of the DRC from https://data.humdata.org/dataset/zones-de-sante-rdc [25]. Source: Global travel-time accessibility indicators.
(TIF)

S10 Fig Travel time to the nearest large port. The green borders correspond to provinces involved in our study. Map produced in Quantum GIS version 3.8.3. using free open shapefiles of the boundaries of the health zones of the DRC from https://data.humdata.org/dataset/zones-de-sante-rdc [25]. Source: Global travel-time accessibility indicators.
(TIF)

S11 Fig Travel time to the nearest medium port. The green borders correspond to provinces involved in our study. Map produced in Quantum GIS version 3.8.3. using free open shapefiles of the boundaries of the health zones of the DRC from https://data.humdata.org/dataset/zones-de-sante-rdc [25]. Source: Global travel-time accessibility indicators.
(TIF)

S12 Fig Travel time to the nearest small port. The green borders correspond to provinces involved in our study. Map produced in Quantum GIS version 3.8.3. using free open shapefiles of the boundaries of the health zones of the DRC from https://data.humdata.org/dataset/zones-de-sante-rdc [25]. Source: Global travel-time accessibility indicators.
(TIF)

S13 Fig Weekly time series of suspected cholera cases reported from 2000 to 2018. Black color corresponds to the two Kivu provinces. Red color corresponds to the endemic areas around Lake Kivu. Source: DRC’s IDSRS.
(TIF)

S14 Fig Weekly time series of types of conflict events reported from 2000 to 2018. Black color corresponds to the two Kivu provinces. Red color corresponds to the areas around Lake Kivu. Source: ACLED.
(TIF)

S15 Fig Impulse response function showing the impact of battles on the number of suspected cholera cases in endemic areas. The highest positive effect of battles on the number of suspected cholera cases is observed in the fourth week. Sources: DRC’s IDSRS and ACLED.
(TIF)

S16 Fig Impulse response function showing the impact of battles on strategic developments in endemic areas. The highest positive effect of battles on strategic developments is observed in the second week. Source: ACLED.
(TIF)

S17 Fig Impulse response function showing the impact of battles on violence against civilians in endemic areas. The highest positive effect of battles on violence against civilians is observed in the second week. Source: ACLED.
(TIF)

S18 Fig Impulse response function showing the impact of battles on the number of IDPs in endemic areas. The highest positive effect of battles on the number of IDPs is observed in the sixth week. Sources: ACLED, and Humanitarian Tools database.
(TIF)

S19 Fig Impulse response function showing the impact of violence against civilians on battles in endemic areas. The highest positive effect of violence against civilians on battles is observed in the first week. Source: ACLED.
(TIF)

S20 Fig Impulse response function showing the impact of violence against civilians on strategic developments in endemic areas. The highest positive effect of violence against civilians on strategic developments is observed in the first week. Source: ACLED.
(TIF)

S21 Fig Impulse response function showing the impact of violence against civilians on the number of IDPs in endemic areas. The highest positive effect of violence against civilians on the number of IDPs is observed in the fourth week. Sources: ACLED and Humanitarian Tools database.
(TIF)

S22 Fig Impulse response function showing the impact of the number of IDPs on riots and protests in endemic areas. The highest positive effect of the number of IDPs on riots and protests is observed in the sixth week. Sources: ACLED and Humanitarian Tools database.
(TIF)

S23 Fig Impulse response function showing the impact of the number of IDPs on violence against civilians in endemic areas. The highest positive effect of the number of IDPs on violence against civilians is observed in the second week. Sources: ACLED and Humanitarian Tools database.
(TIF)

S24 Fig Impulse response function showing the impact of strategic developments on violence against civilians in endemic areas. The highest positive effect of strategic developments on violence against civilians is observed in the first week. Source: ACLED.
(TIF)

S25 Fig Impulse response function showing the impact of riots and protests on the number of IDPs in endemic areas. The highest positive effect of riots and protests on the number of IDPs is in the eighth week. Sources: ACLED and Humanitarian Tools database.
(TIF)

S26 Fig Impulse response function showing the impact of battles on violence against civilians in non-endemic areas. The highest positive effect of battles on violence against civilians is observed in the first week. Source: ACLED.
(TIF)

S27 Fig Impulse response function showing the impact of battles on the number of IDPs in non-endemic areas. The highest positive effect of battles on the number of IDPs is observed in the fourth week. Sources: ACLED and Humanitarian Tools database.
(TIF)

S28 Fig Impulse response function showing the impact of battles on strategic developments in non-endemic areas. The highest positive effect of battles on strategic developments is observed in the first week. Source: ACLED.
(TIF)

S29 Fig Impulse response function showing the impact of violence against civilians on battles in non-endemic areas. The highest positive effect of violence against civilians on battles is observed in the first week. Source: ACLED.
(TIF)

S30 Fig Impulse response function showing the impact of violence against civilians on the number of IDPs in non-endemic areas. The highest positive effect of violence against civilians on the number of IDPs is observed in the first week. Sources: ACLED and Humanitarian Tools database.
(TIF)

S31 Fig Impulse response function showing the impact of violence against civilians on riots and protests in non-endemic areas. The highest positive effect of violence against civilians on riots and protests is observed in the seventh week. Source: ACLED.
(TIF)

S32 Fig Impulse response function showing the impact of the number of IDPs on battles in non-endemic areas. The highest positive effect of the number of IDPs on battles is observed in the fourth week. Sources: ACLED and Humanitarian Tools database.
(TIF)

S33 Fig Impulse response function showing the impact of the number of IDPs on violence against civilians in non-endemic areas. The highest positive effect of the number of IDPs on violence against civilians is observed in the fourth week. Sources: ACLED and Humanitarian Tools database.
(TIF)

S34 Fig Impulse response function showing the impact of the number of IDPs on riots and protests in non-endemic areas. The highest positive effect of the number of IDPs on riots and protests is observed in the eighth week. Sources: ACLED and Humanitarian Tools database.
(TIF)

S35 Fig Impulse response function showing the impact of riots and protests on the number of IDPs in non-endemic areas. The highest positive effect of riots and protests on the number of IDPs is observed in the eighth week. Sources: ACLED and Humanitarian Tools database.
(TIF)

S36 Fig Impulse response function showing the impact of riots and protests on violence against civilians in non-endemic areas. The highest positive effect of riots and protests on violence against civilians is observed in the eighth week. Source: ACLED.
(TIF)

S37 Fig Impulse response function showing the impact of strategic developments on battles in non-endemic areas. The highest positive effect of strategic developments on battles is observed in the first week. Source: ACLED.
(TIF)

S38 Fig Comparison of characteristics using Kruskal-Wallis test. Sources: DRC’s IDSRS, WorldPop, Humanitarian Data Exchange, and Global travel-time accessibility indicators.
(TIF)

S39 Fig Distribution of households using drinking water from improved sources at the provincial level. The green borders correspond to provinces involved in our study. Map produced in Quantum GIS version 3.8.3. using free open shapefiles of the first level administrative boundaries of the DRC from https://data.humdata.org/dataset/wfp-geonode-drc-first-level-administrative-boundaries. Source: Multiple Indicator Cluster Survey.
(TIF)

S40 Fig Provincial distribution of households with handwashing facilities where soap and water are present. The green borders correspond to provinces involved in our study. Map produced in Quantum GIS version 3.8.3. using free open shapefiles of the first level administrative boundaries of the DRC from https://data.humdata.org/dataset/wfp-geonode-drc-first-level-administrative-boundaries. Source: Multiple Indicator Cluster Survey.
(TIF)

S41 Fig Distribution of households using sanitation facilities at the provincial level. The green borders correspond to provinces involved in our study. Map produced in Quantum GIS version 3.8.3. using free open shapefiles of the first level administrative boundaries of the DRC from https://data.humdata.org/dataset/wfp-geonode-drc-first-level-administrative-boundaries. Source: Multiple Indicator Cluster Survey.
(TIF)

S42 Fig Provincial distribution of open defecation. The green borders correspond to provinces involved in our study. Map produced in Quantum GIS version 3.8.3. using free open shapefiles of the first level administrative boundaries of the DRC from https://data.humdata.org/dataset/wfp-geonode-drc-first-level-administrative-boundaries. Source: Multiple Indicator Cluster Survey.
(TIF)

S1 Table Annual number of suspected cholera cases reported in the DRC and Kivu provinces, 2000–2018. Source: DRC’s IDSRS.
(DOCX)

S2 Table Summary of conflict events reported in the DRC, 2000–2018. Source: ACLED. (DOCX)

S3 Table Summary of types of conflict events reported by provinces, 2000–2018. Source: ACLED.
(DOCX)

S4 Table Summary of types of conflict events reported in the Kivu provinces, and areas bordering Lake Kivu, 2000–2018. Source: ACLED.
(DOCX)

S5 Table Summary of types of conflict events reported in areas around those bordering Lake Kivu according to cholera status, 2000–2018. Source: ACLED.
(DOCX)

S6 Table Summary of the number of IDPs reported by provinces, 2009–2018. Source: Humanitarian Tools database.
(DOCX)

S7 Table Summary of the number of IDPs reported in the Kivu provinces, and cholera endemic areas bordering Lake Kivu, 2009–2018. Source: Humanitarian Tools database.
(DOCX)

S8 Table Summary of the number of IDPs reported in areas around those bordering Lake Kivu according to cholera status, 2009–2018. Source: Humanitarian Tools database.
(DOCX)

S1 Dataset Database for spatial clustering analysis. (TXT)

S2 Dataset Database for the modelling framework. (TXT)

S3 Dataset Database for classification of the health zones affected by cholera epidemic waves. (TXT)
