# Supplementary figures and images for: Drivers of the dynamics of the spread of cholera in the Democratic Republic of the Congo, 2000–2018: An eco-epidemiological study
Source: PLoS Negl Trop Dis. 2023 Aug 28;17(8):e0011597. doi: 10.1371/journal.pntd.0011597 (PMC10491302; doi:10.1371/journal.pntd.0011597)

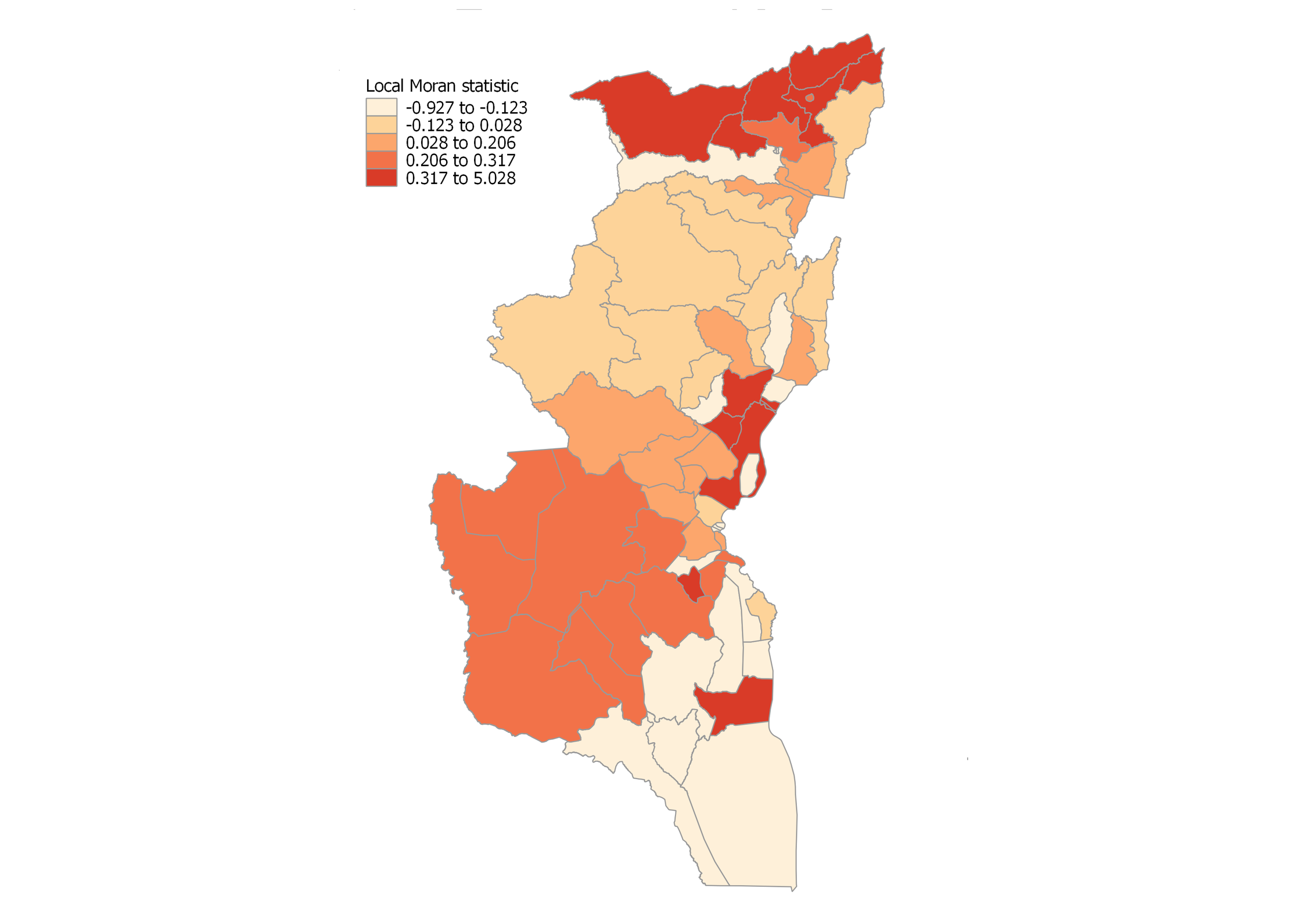

Supplement: S1 Fig — Source: DRC’s IDSRS. (TIF) [file pntd.0011597.s001.tif]

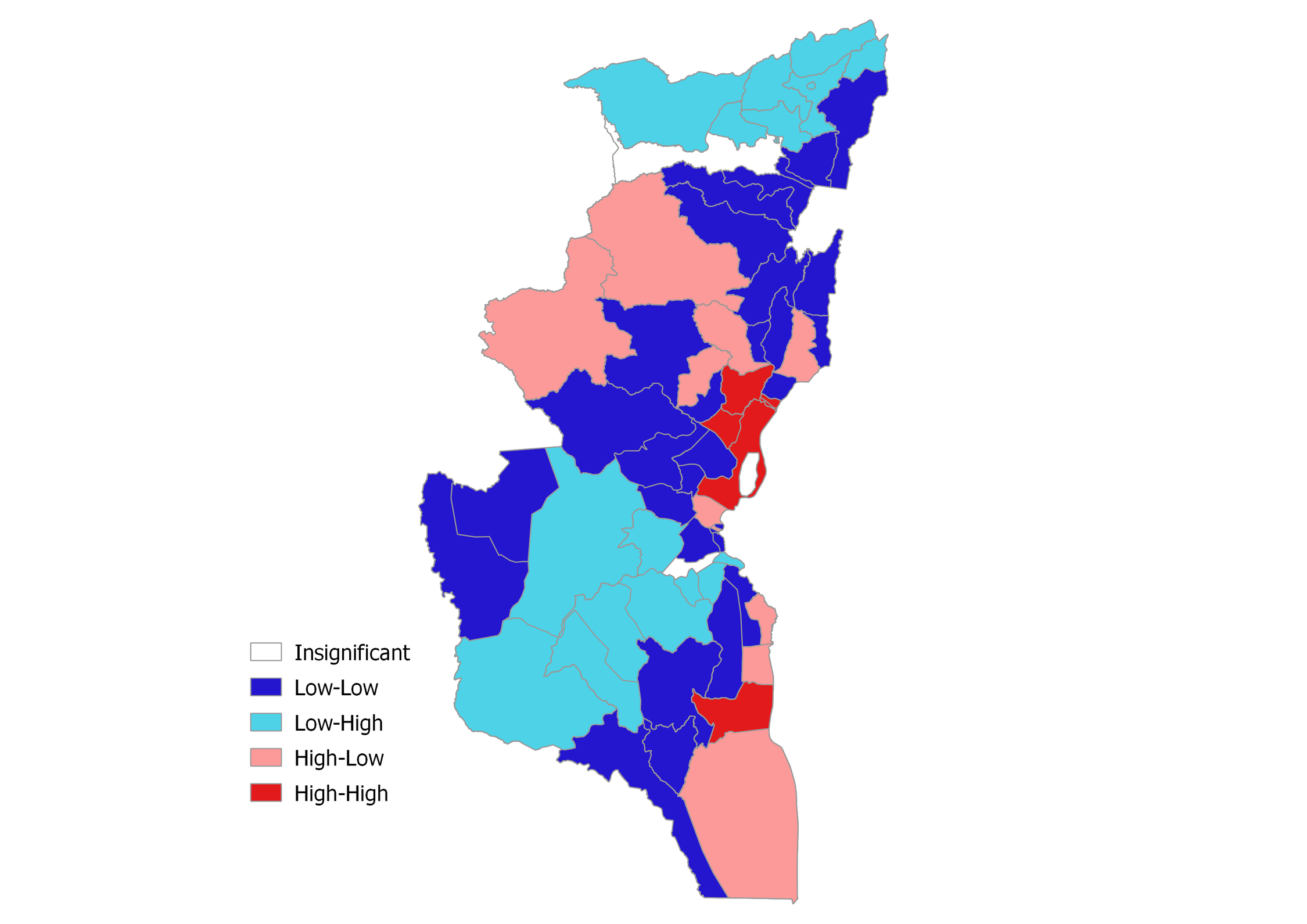

Supplement: S2 Fig — Source: DRC’s IDSRS. (TIF) [file pntd.0011597.s002.tif]

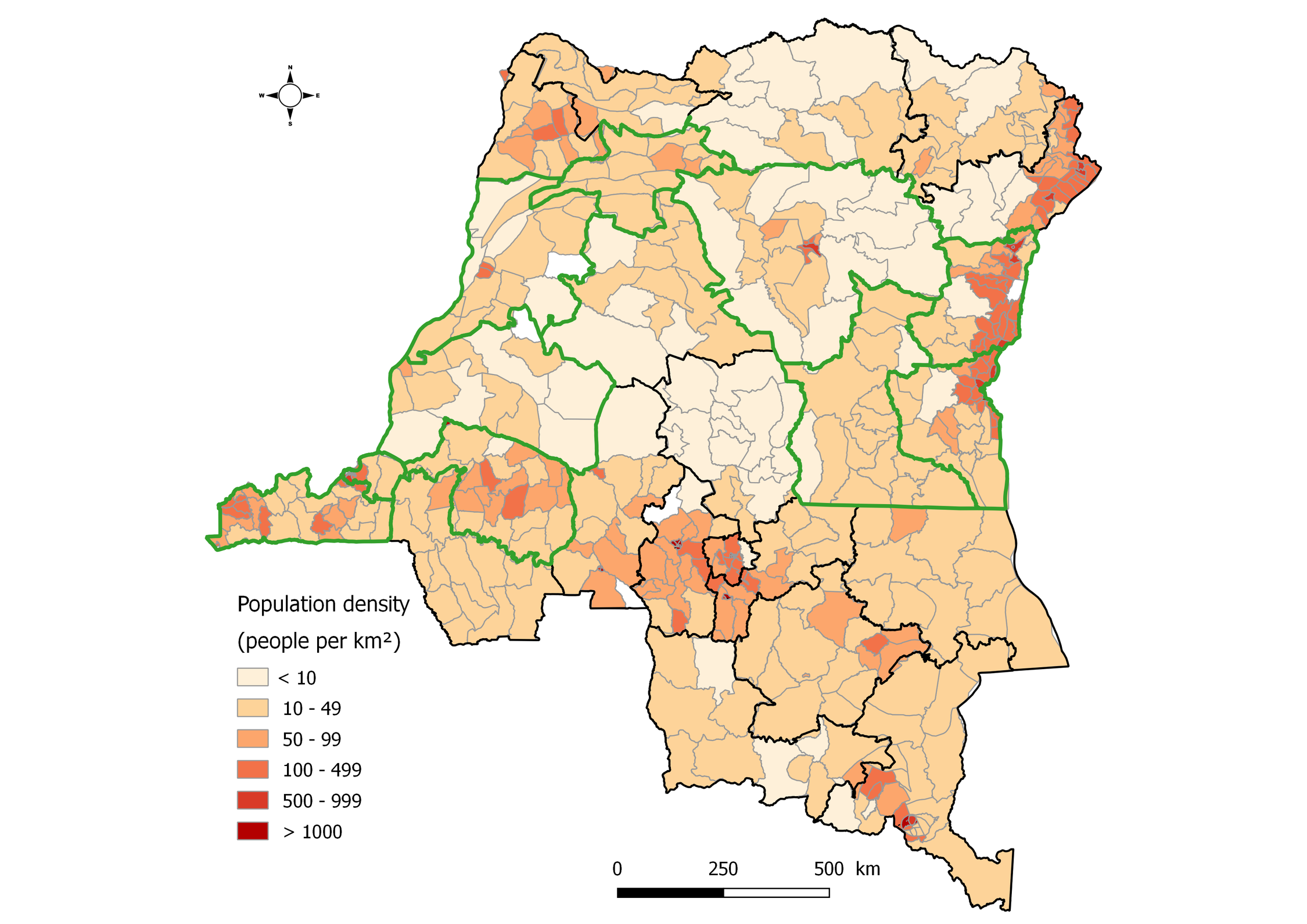

Supplement: S3 Fig — The green borders correspond to provinces involved in our study. Map produced in Quantum GIS version 3.8.3. using free open shapefiles of the boundaries of the health zones of the DRC from https://data.humdata.org/dataset/zones-de-sante-rdc [25]. Source: Worldpop. (TIF) [file pntd.0011597.s003.tif]

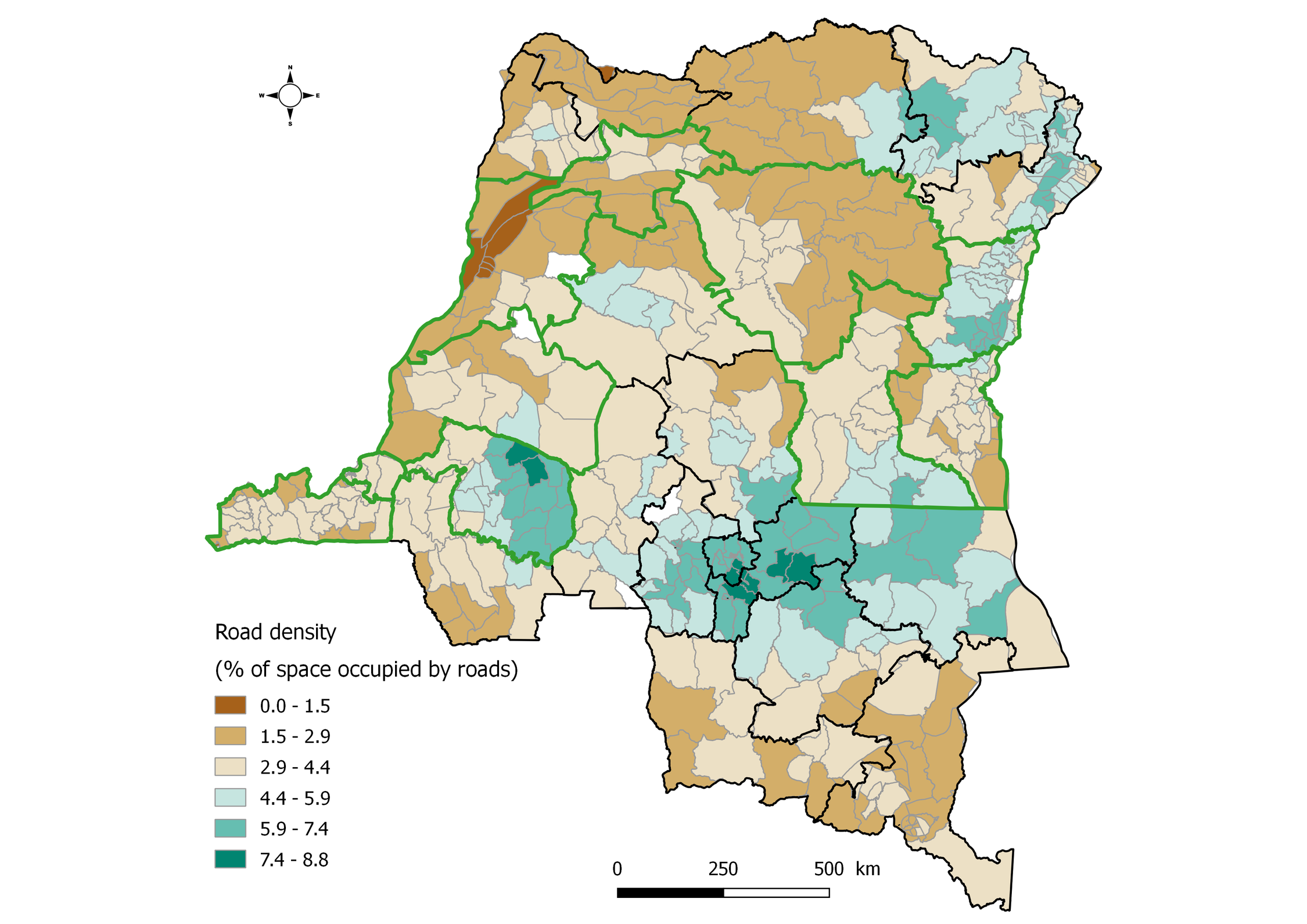

Supplement: S4 Fig — The green borders correspond to provinces involved in our study. Map produced in Quantum GIS version 3.8.3. using free open shapefiles of the boundaries of the health zones of the DRC from https://data.humdata.org/dataset/zones-de-sante-rdc [25]. Source: Humanitarian Data Exchange. (TIF) [file pntd.0011597.s004.tif]

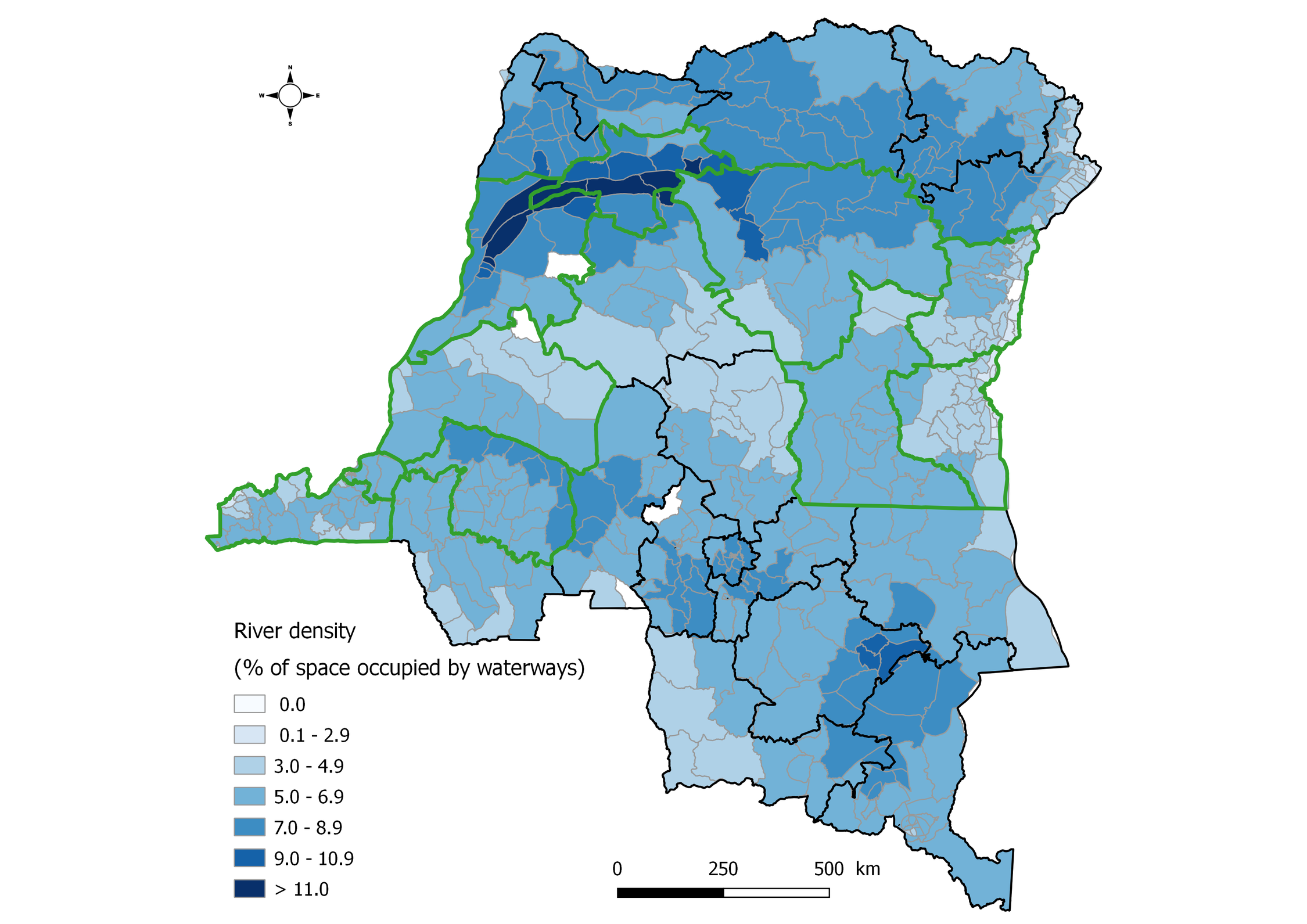

Supplement: S5 Fig — The green borders correspond to provinces involved in our study. Map produced in Quantum GIS version 3.8.3. using free open shapefiles of the boundaries of the health zones of the DRC from https://data.humdata.org/dataset/zones-de-sante-rdc [25]. Source: Humanitarian Data Exchange. (TIF) [file pntd.0011597.s005.tif]

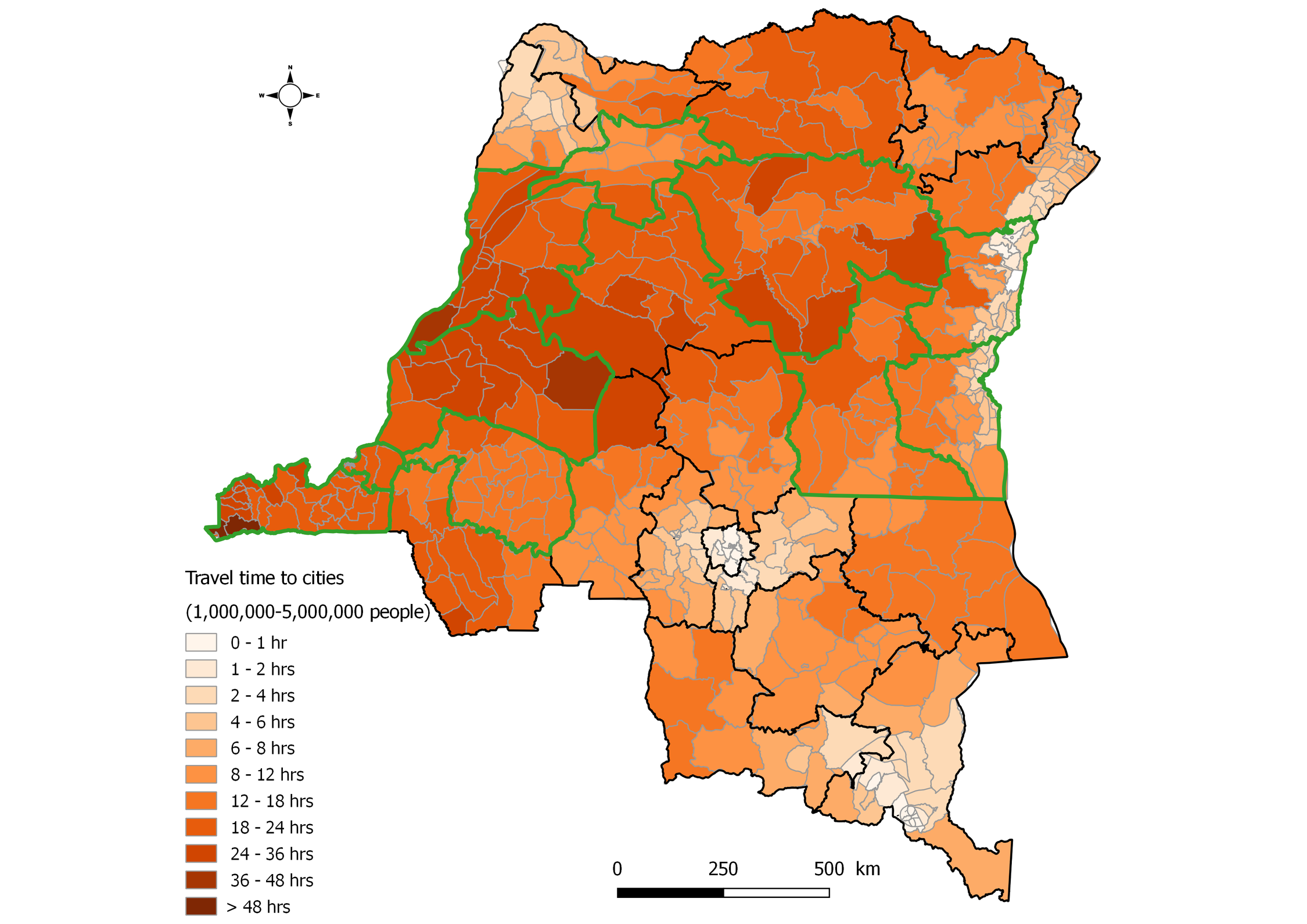

Supplement: S6 Fig — The green borders correspond to provinces involved in our study. Map produced in Quantum GIS version 3.8.3. using free open shapefiles of the boundaries of the health zones of the DRC from https://data.humdata.org/dataset/zones-de-sante-rdc [25]. Source: Global travel-time accessibility indicators. (TIF) [file pntd.0011597.s006.tif]

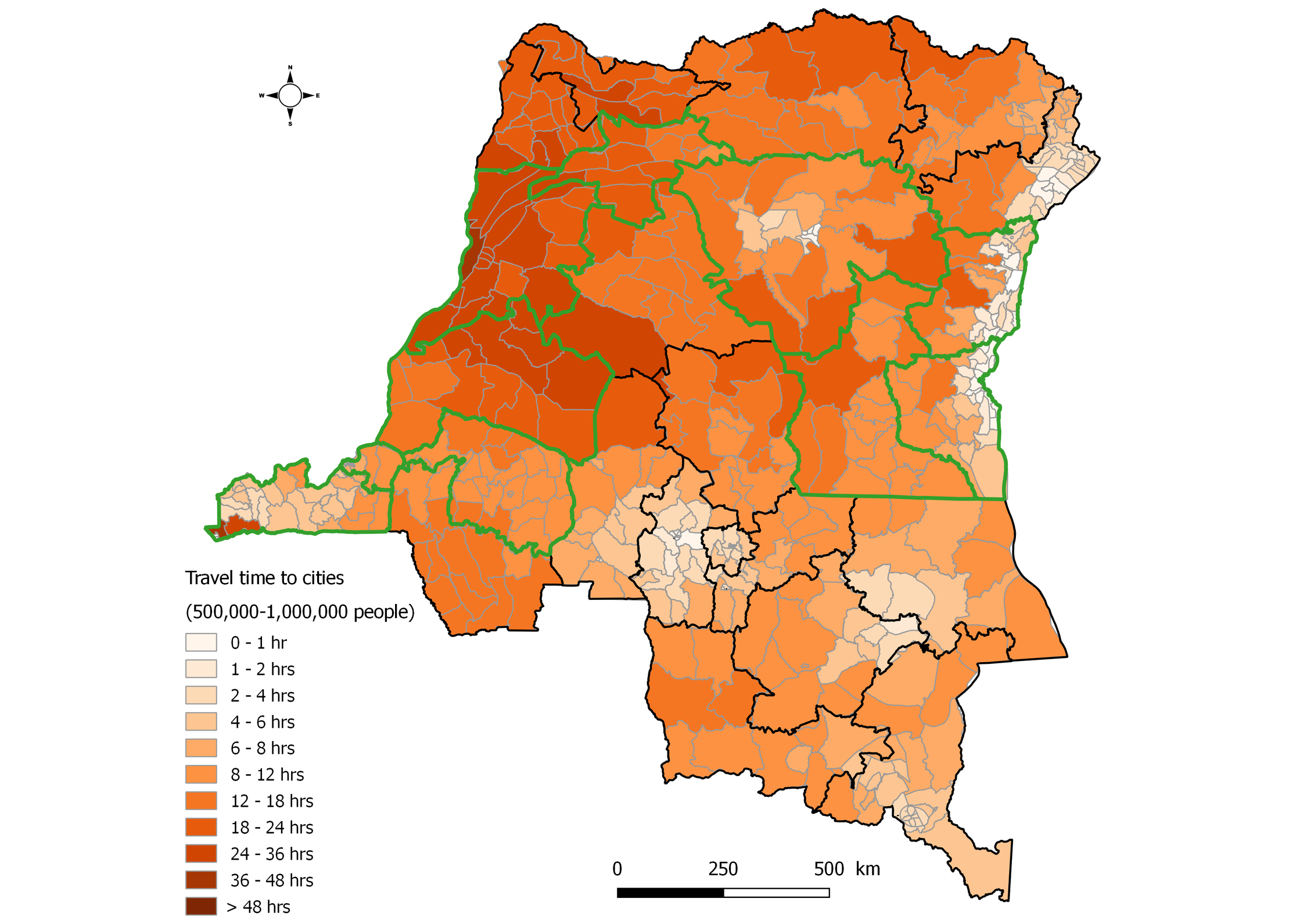

Supplement: S7 Fig — The green borders correspond to provinces involved in our study. Map produced in Quantum GIS version 3.8.3. using free open shapefiles of the boundaries of the health zones of the DRC from https://data.humdata.org/dataset/zones-de-sante-rdc [25]. Source: Global travel-time accessibility indicators. (TIF) [file pntd.0011597.s007.tif]

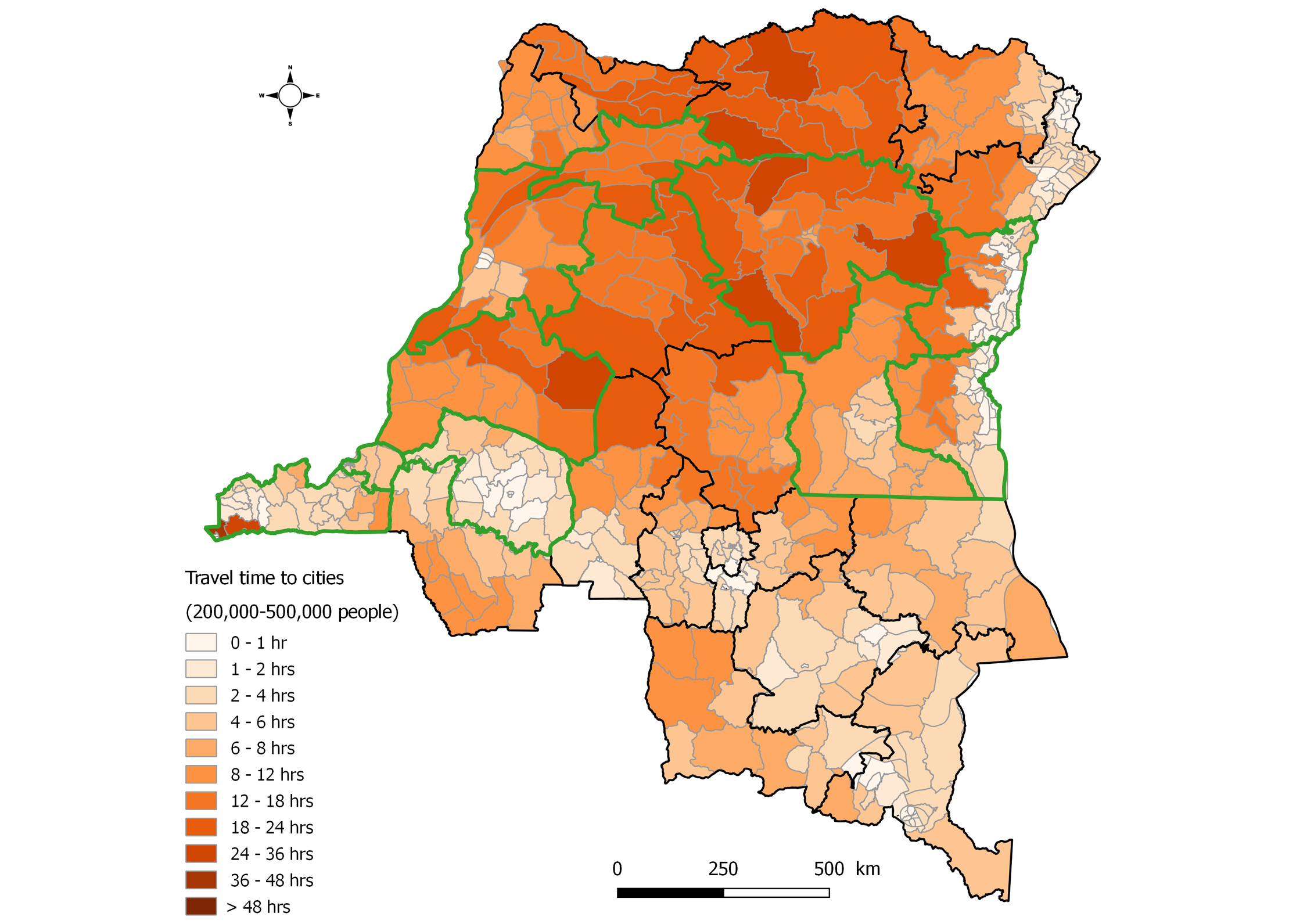

Supplement: S8 Fig — The green borders correspond to provinces involved in our study. Map produced in Quantum GIS version 3.8.3. using free open shapefiles of the boundaries of the health zones of the DRC from https://data.humdata.org/dataset/zones-de-sante-rdc [25]. Source: Global travel-time accessibility indicators. (TIF) [file pntd.0011597.s008.tif]

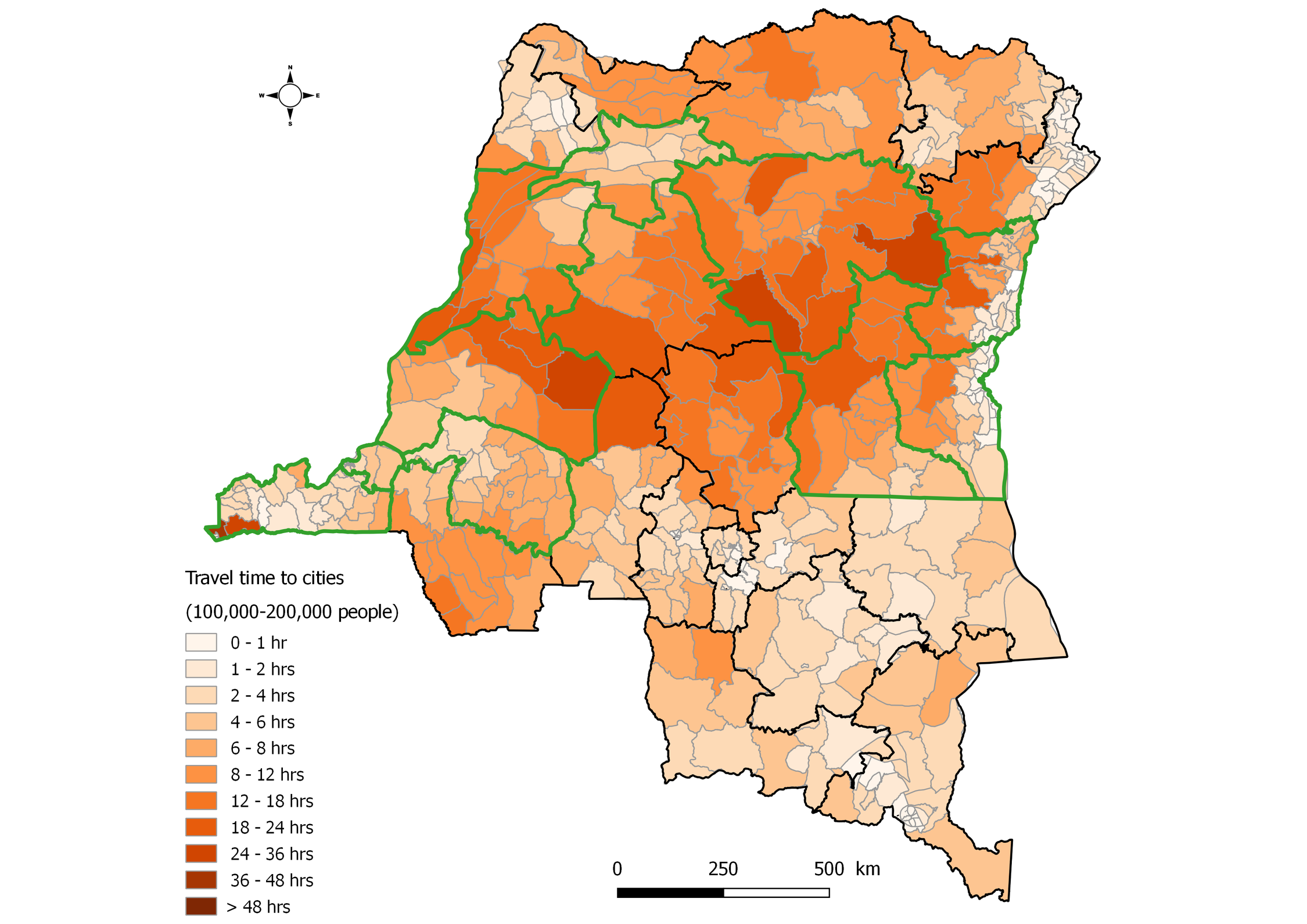

Supplement: S9 Fig — The green borders correspond to provinces involved in our study. Map produced in Quantum GIS version 3.8.3. using free open shapefiles of the boundaries of the health zones of the DRC from https://data.humdata.org/dataset/zones-de-sante-rdc [25]. Source: Global travel-time accessibility indicators. (TIF) [file pntd.0011597.s009.tif]

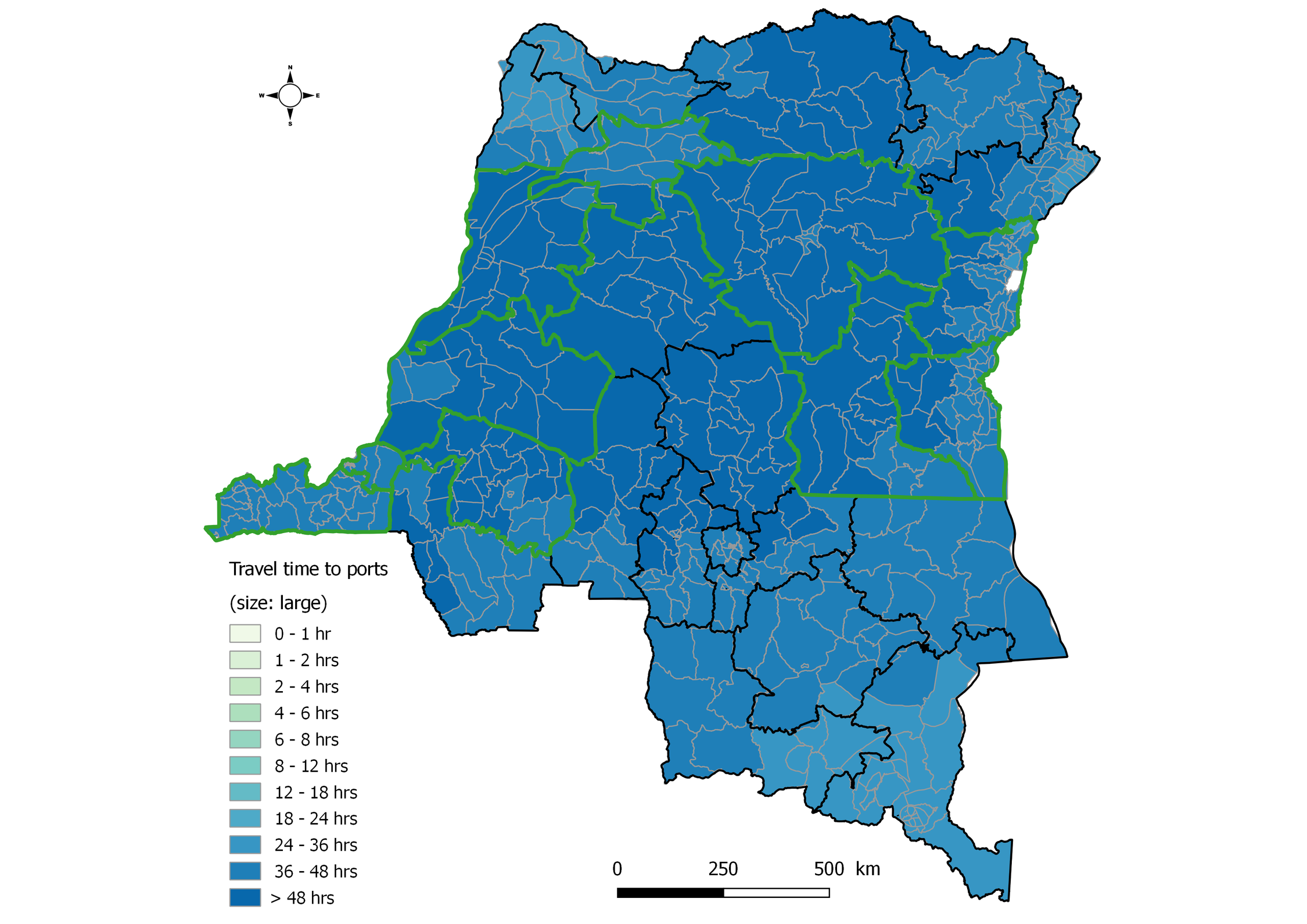

Supplement: S10 Fig — The green borders correspond to provinces involved in our study. Map produced in Quantum GIS version 3.8.3. using free open shapefiles of the boundaries of the health zones of the DRC from https://data.humdata.org/dataset/zones-de-sante-rdc [25]. Source: Global travel-time accessibility indicators. (TIF) [file pntd.0011597.s010.tif]

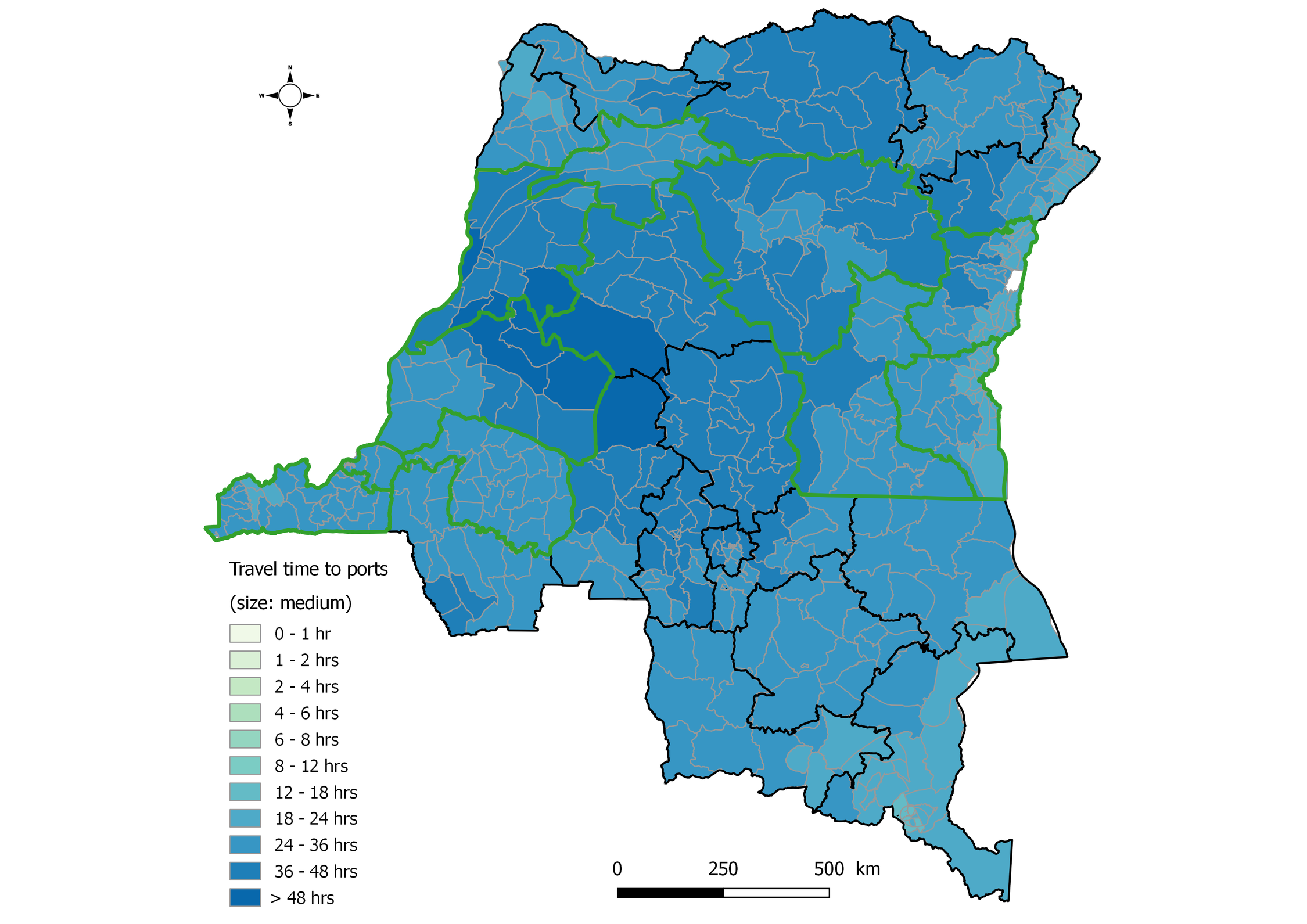

Supplement: S11 Fig — The green borders correspond to provinces involved in our study. Map produced in Quantum GIS version 3.8.3. using free open shapefiles of the boundaries of the health zones of the DRC from https://data.humdata.org/dataset/zones-de-sante-rdc [25]. Source: Global travel-time accessibility indicators. (TIF) [file pntd.0011597.s011.tif]

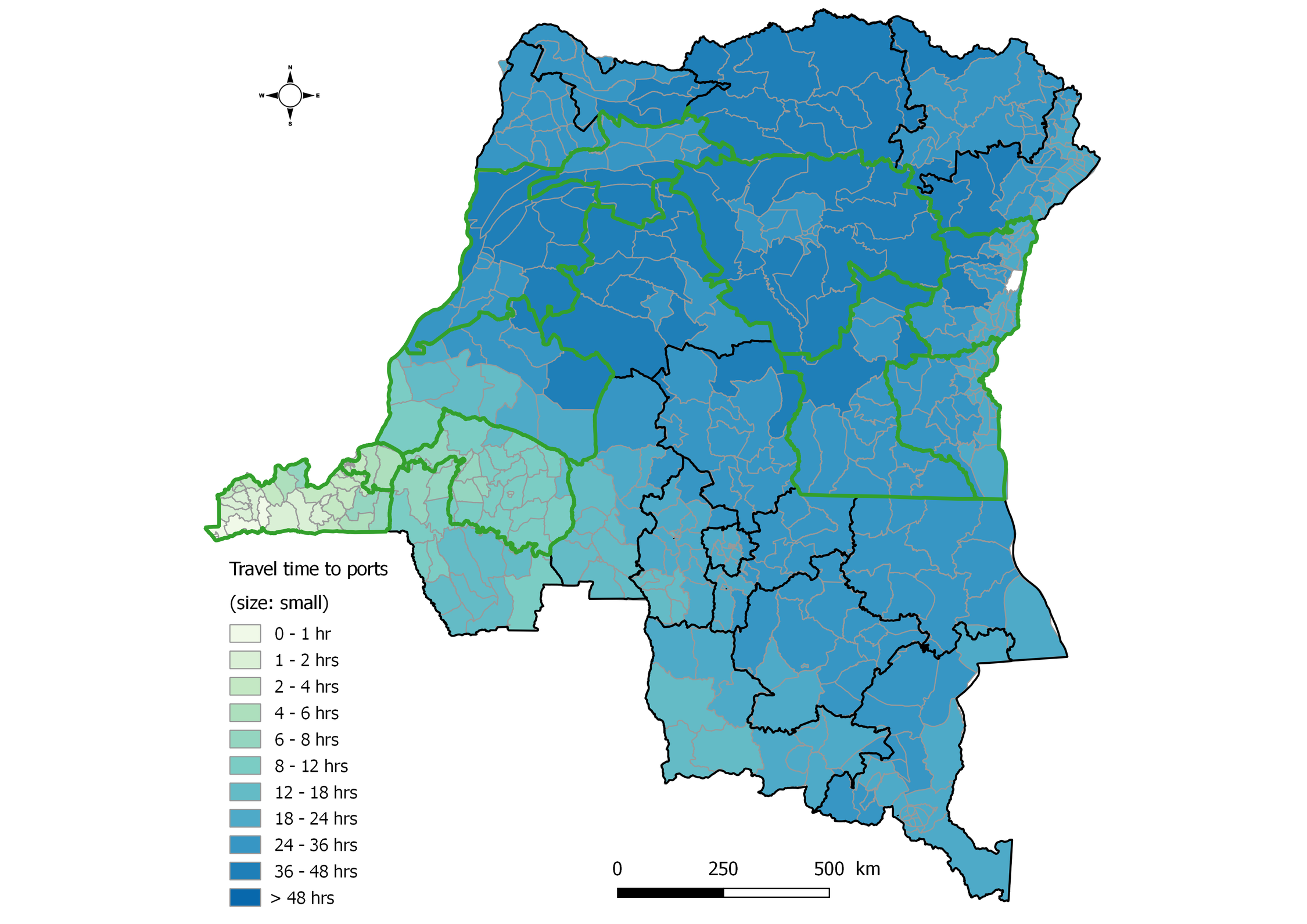

Supplement: S12 Fig — The green borders correspond to provinces involved in our study. Map produced in Quantum GIS version 3.8.3. using free open shapefiles of the boundaries of the health zones of the DRC from https://data.humdata.org/dataset/zones-de-sante-rdc [25]. Source: Global travel-time accessibility indicators. (TIF) [file pntd.0011597.s012.tif]

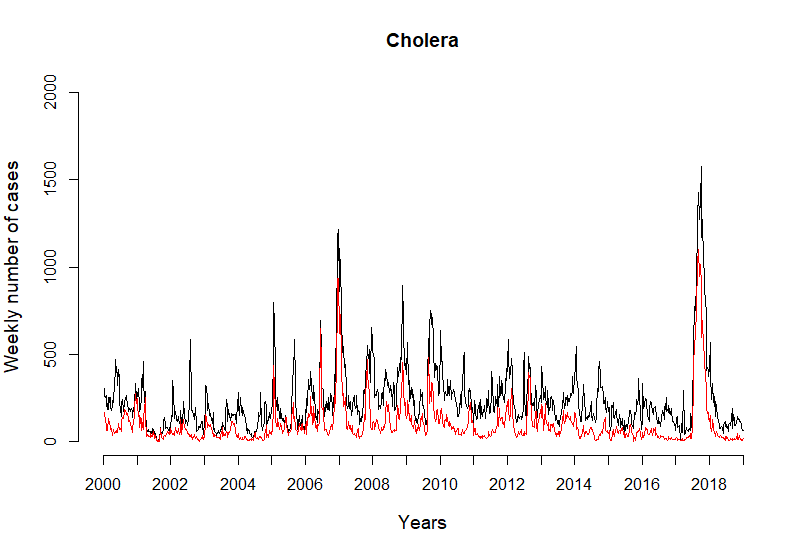

Supplement: S13 Fig — Black color corresponds to the two Kivu provinces. Red color corresponds to the endemic areas around Lake Kivu. Source: DRC’s IDSRS. (TIF) [file pntd.0011597.s013.tif]

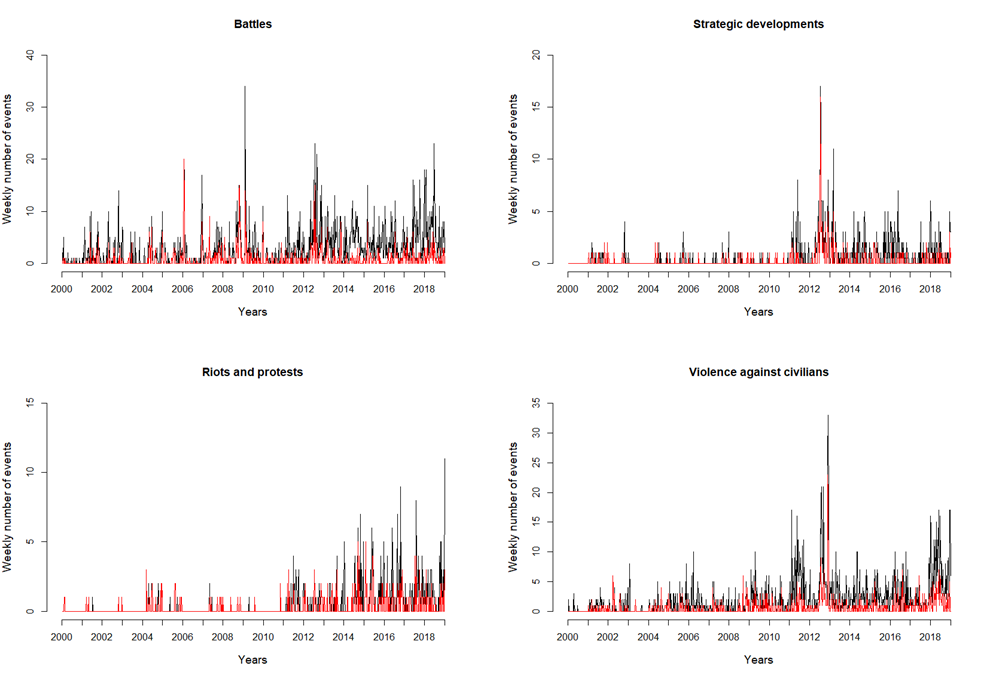

Supplement: S14 Fig — Black color corresponds to the two Kivu provinces. Red color corresponds to the areas around Lake Kivu. Source: ACLED. (TIF) [file pntd.0011597.s014.tif]

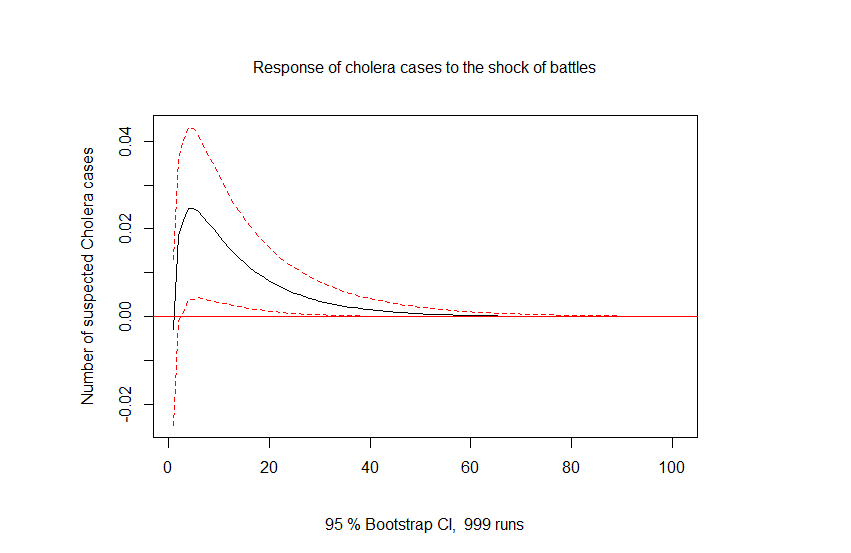

Supplement: S15 Fig — The highest positive effect of battles on the number of suspected cholera cases is observed in the fourth week. Sources: DRC’s IDSRS and ACLED. (TIF) [file pntd.0011597.s015.tif]

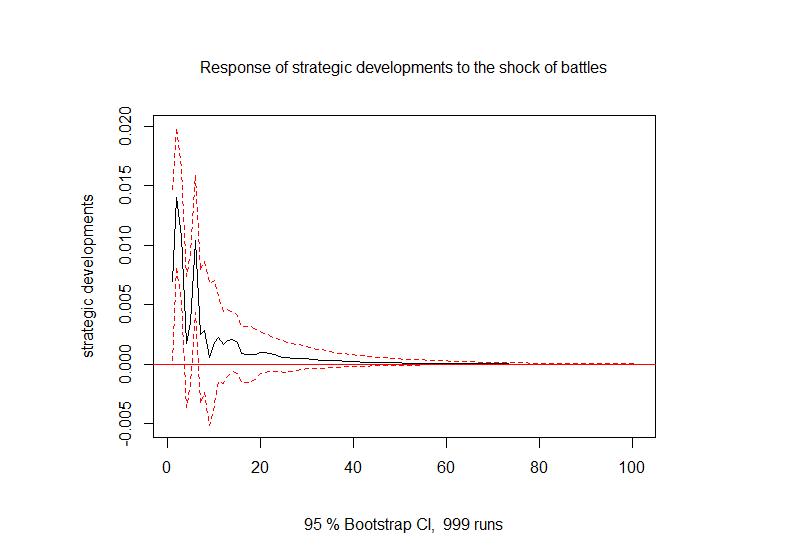

Supplement: S16 Fig — The highest positive effect of battles on strategic developments is observed in the second week. Source: ACLED. (TIF) [file pntd.0011597.s016.tif]

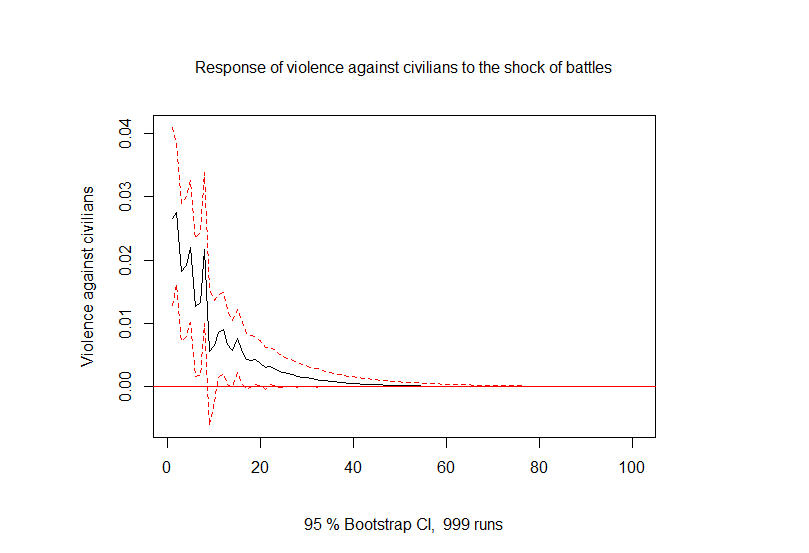

Supplement: S17 Fig — The highest positive effect of battles on violence against civilians is observed in the second week. Source: ACLED. (TIF) [file pntd.0011597.s017.tif]

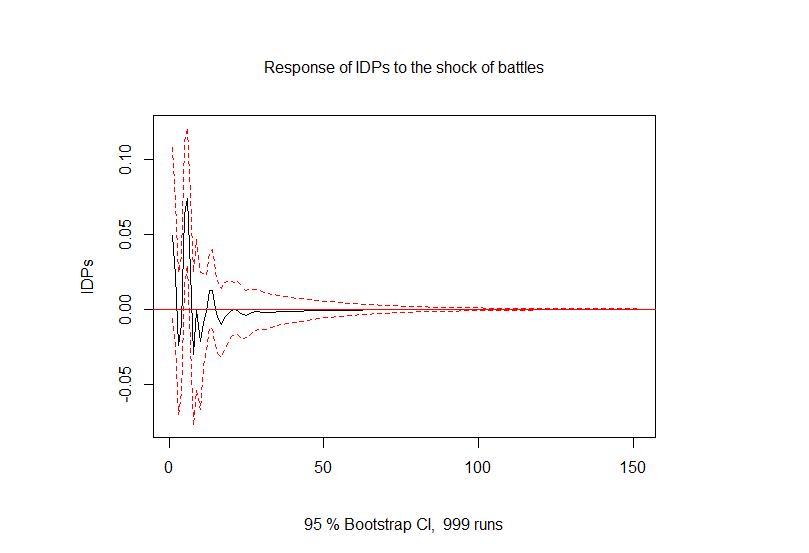

Supplement: S18 Fig — The highest positive effect of battles on the number of IDPs is observed in the sixth week. Sources: ACLED, and Humanitarian Tools database. (TIF) [file pntd.0011597.s018.tif]

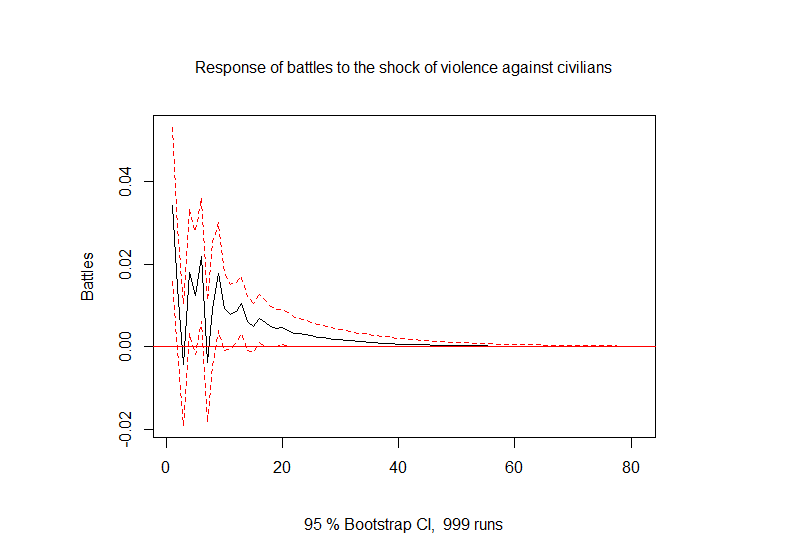

Supplement: S19 Fig — The highest positive effect of violence against civilians on battles is observed in the first week. Source: ACLED. (TIF) [file pntd.0011597.s019.tif]

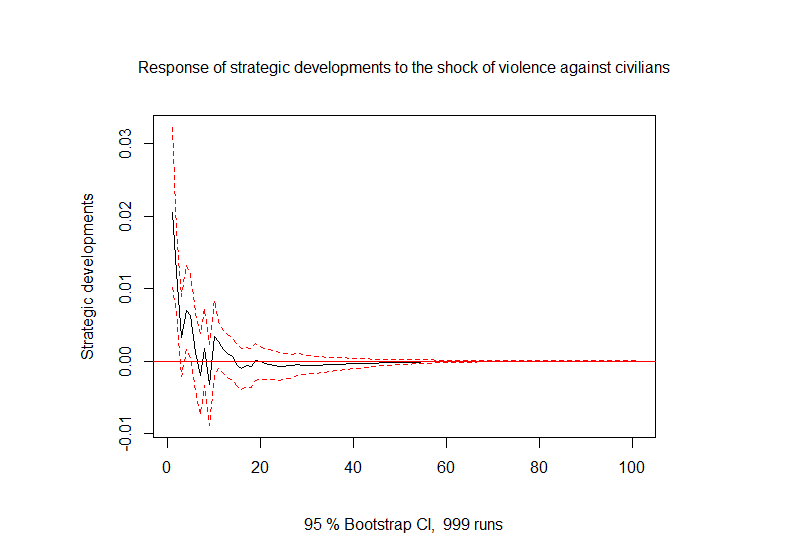

Supplement: S20 Fig — The highest positive effect of violence against civilians on strategic developments is observed in the first week. Source: ACLED. (TIF) [file pntd.0011597.s020.tif]

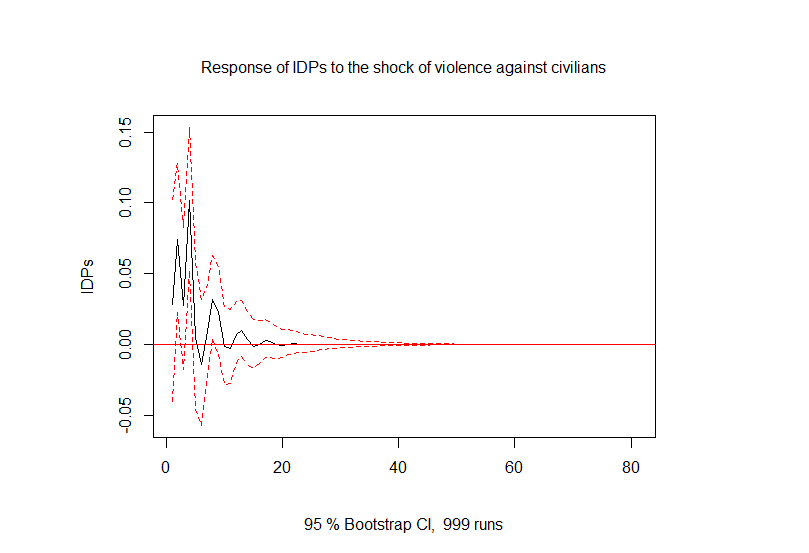

Supplement: S21 Fig — The highest positive effect of violence against civilians on the number of IDPs is observed in the fourth week. Sources: ACLED and Humanitarian Tools database. (TIF) [file pntd.0011597.s021.tif]

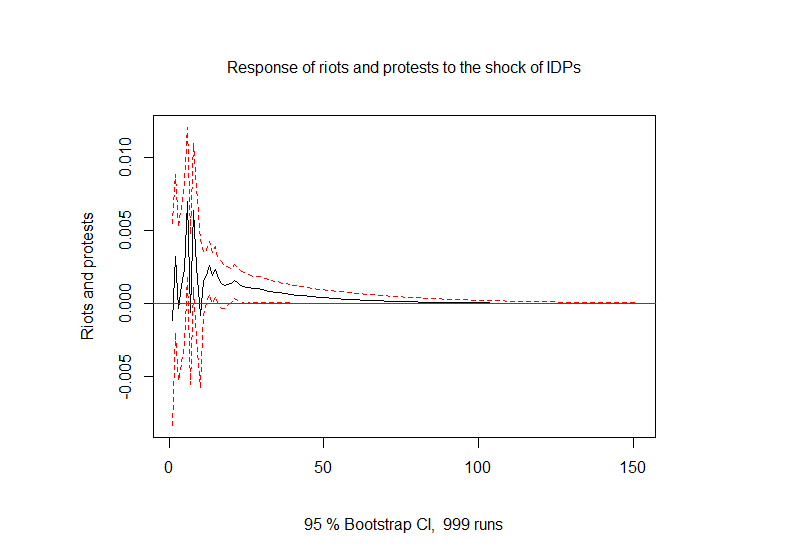

Supplement: S22 Fig — The highest positive effect of the number of IDPs on riots and protests is observed in the sixth week. Sources: ACLED and Humanitarian Tools database. (TIF) [file pntd.0011597.s022.tif]

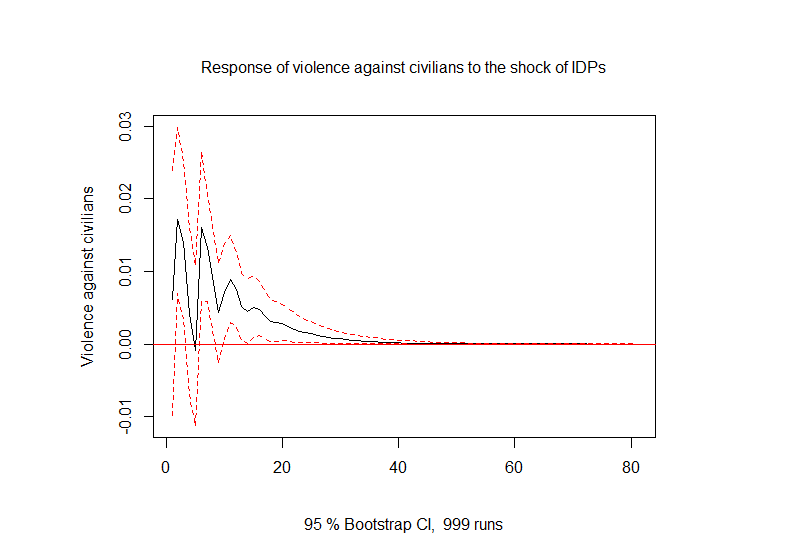

Supplement: S23 Fig — The highest positive effect of the number of IDPs on violence against civilians is observed in the second week. Sources: ACLED and Humanitarian Tools database. (TIF) [file pntd.0011597.s023.tif]

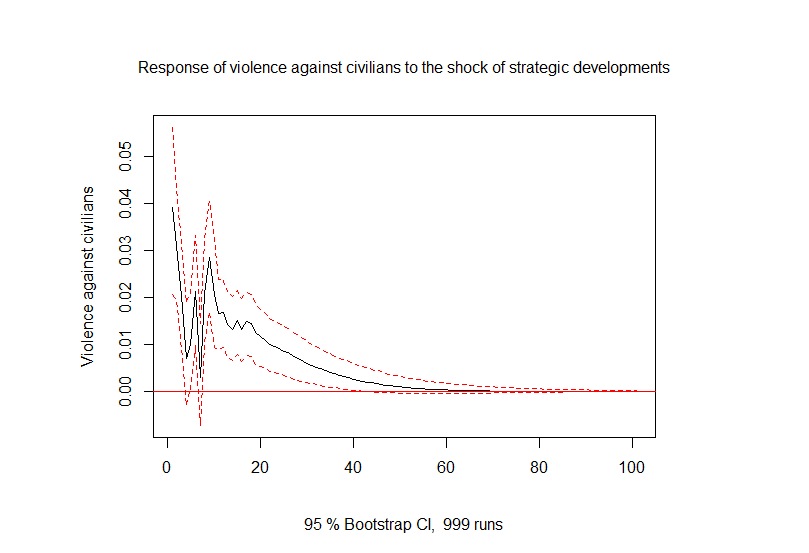

Supplement: S24 Fig — The highest positive effect of strategic developments on violence against civilians is observed in the first week. Source: ACLED. (TIF) [file pntd.0011597.s024.tif]

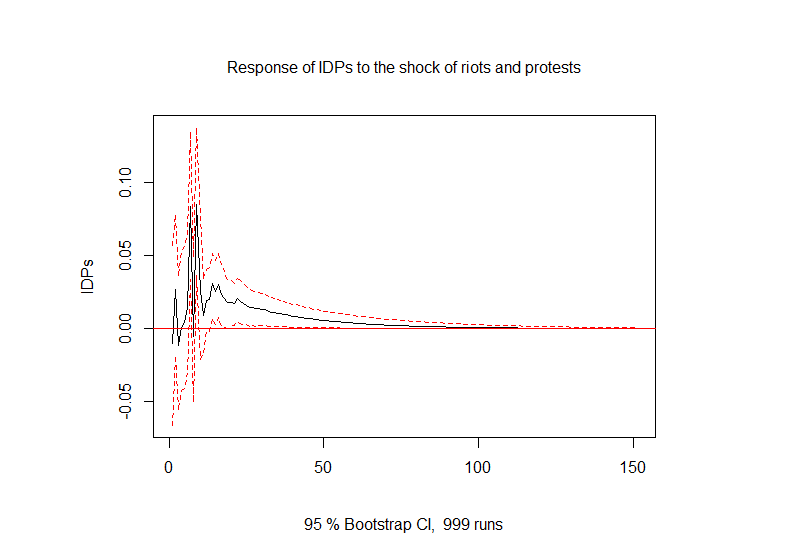

Supplement: S25 Fig — The highest positive effect of riots and protests on the number of IDPs is in the eighth week. Sources: ACLED and Humanitarian Tools database. (TIF) [file pntd.0011597.s025.tif]

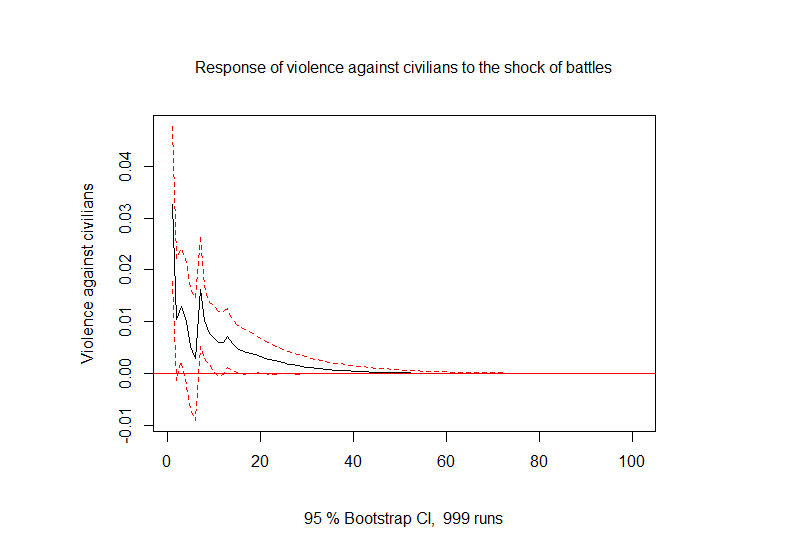

Supplement: S26 Fig — The highest positive effect of battles on violence against civilians is observed in the first week. Source: ACLED. (TIF) [file pntd.0011597.s026.tif]

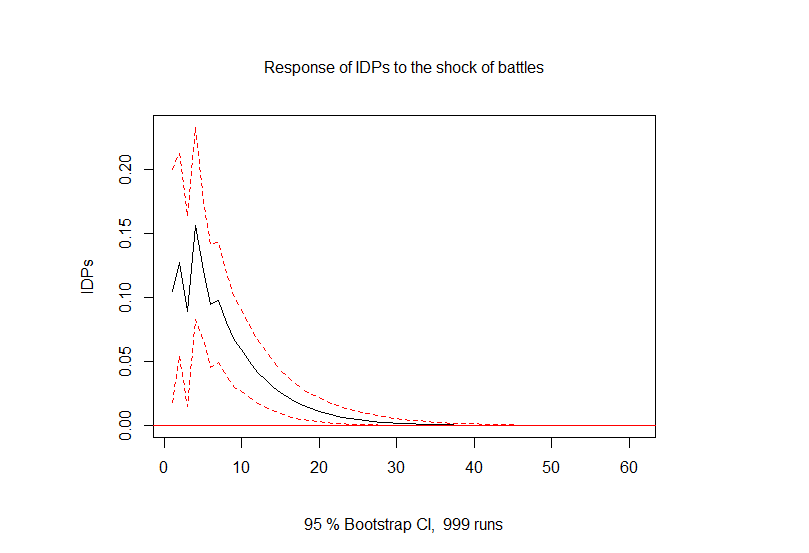

Supplement: S27 Fig — The highest positive effect of battles on the number of IDPs is observed in the fourth week. Sources: ACLED and Humanitarian Tools database. (TIF) [file pntd.0011597.s027.tif]

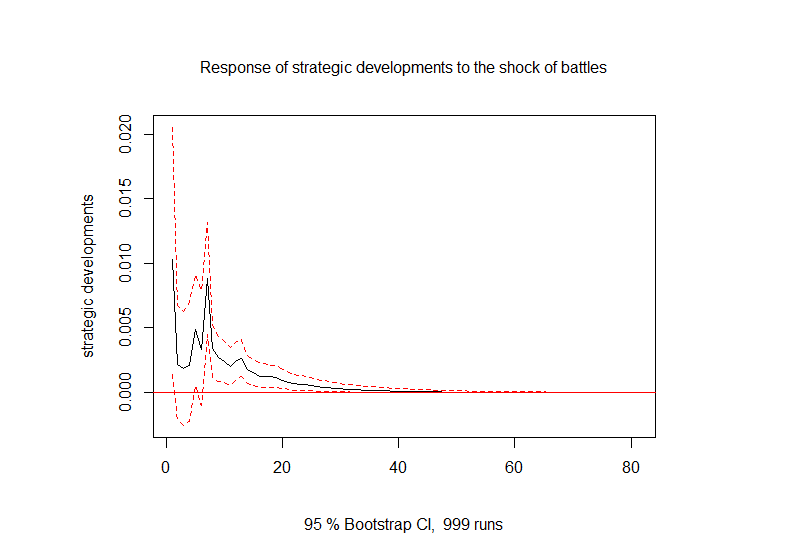

Supplement: S28 Fig — The highest positive effect of battles on strategic developments is observed in the first week. Source: ACLED. (TIF) [file pntd.0011597.s028.tif]

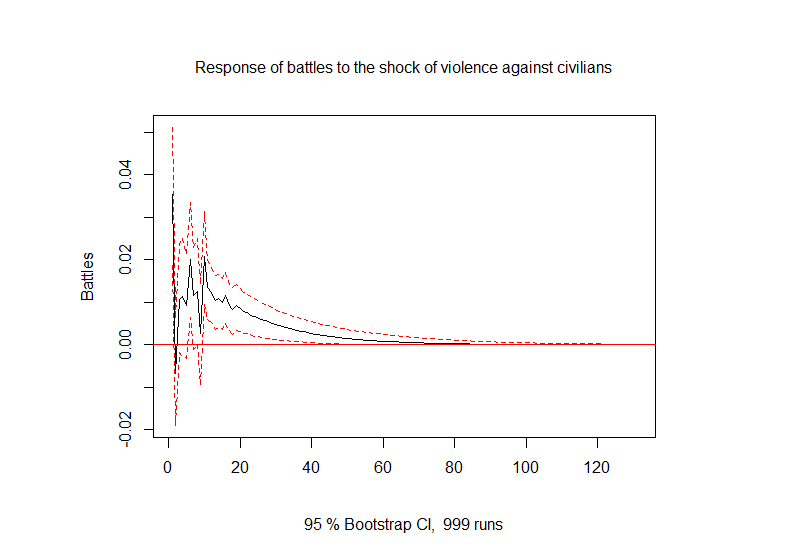

Supplement: S29 Fig — The highest positive effect of violence against civilians on battles is observed in the first week. Source: ACLED. (TIF) [file pntd.0011597.s029.tif]

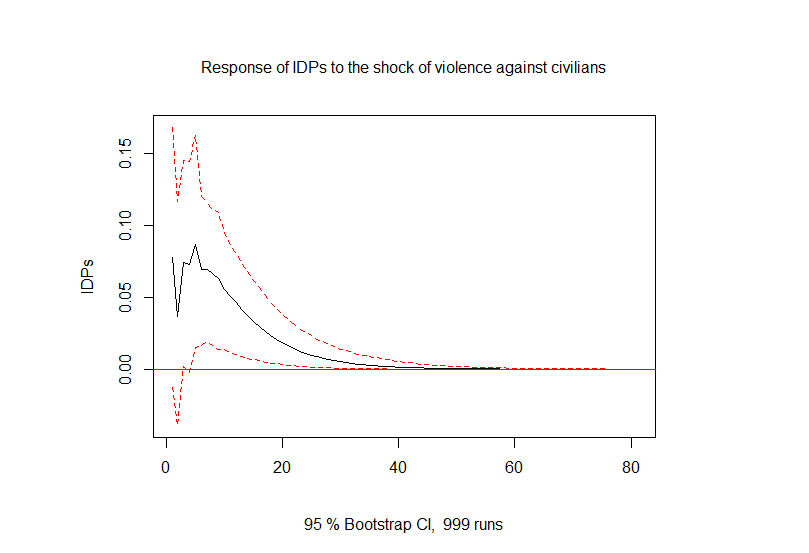

Supplement: S30 Fig — The highest positive effect of violence against civilians on the number of IDPs is observed in the first week. Sources: ACLED and Humanitarian Tools database. (TIF) [file pntd.0011597.s030.tif]

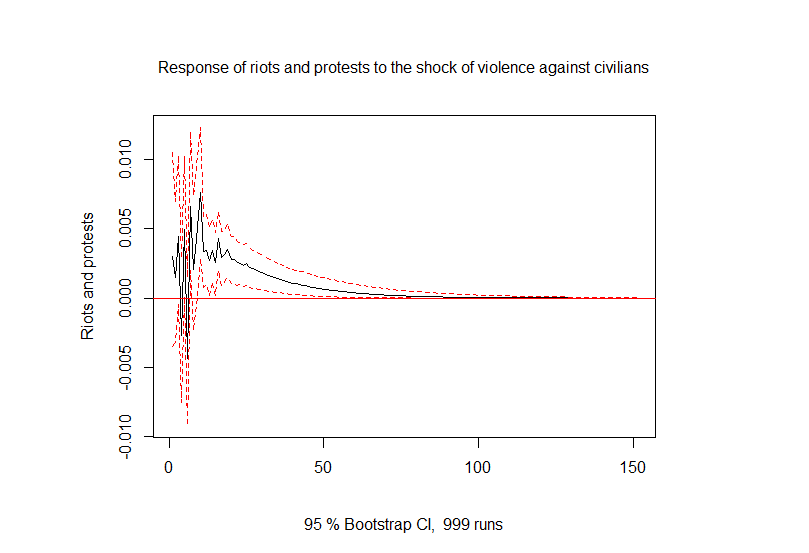

Supplement: S31 Fig — The highest positive effect of violence against civilians on riots and protests is observed in the seventh week. Source: ACLED. (TIF) [file pntd.0011597.s031.tif]

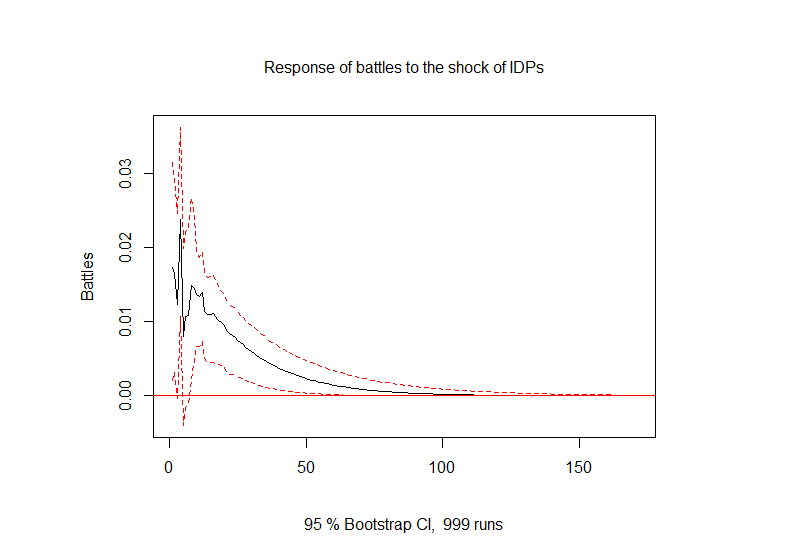

Supplement: S32 Fig — The highest positive effect of the number of IDPs on battles is observed in the fourth week. Sources: ACLED and Humanitarian Tools database. (TIF) [file pntd.0011597.s032.tif]

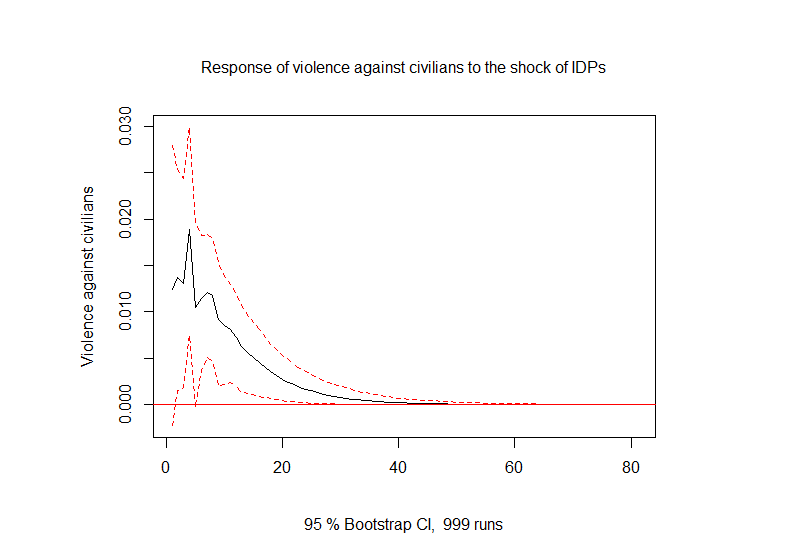

Supplement: S33 Fig — The highest positive effect of the number of IDPs on violence against civilians is observed in the fourth week. Sources: ACLED and Humanitarian Tools database. (TIF) [file pntd.0011597.s033.tif]

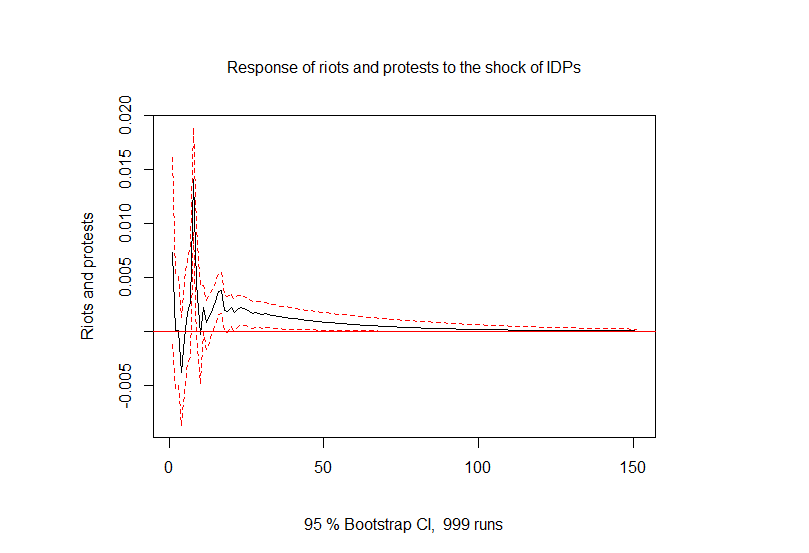

Supplement: S34 Fig — The highest positive effect of the number of IDPs on riots and protests is observed in the eighth week. Sources: ACLED and Humanitarian Tools database. (TIF) [file pntd.0011597.s034.tif]

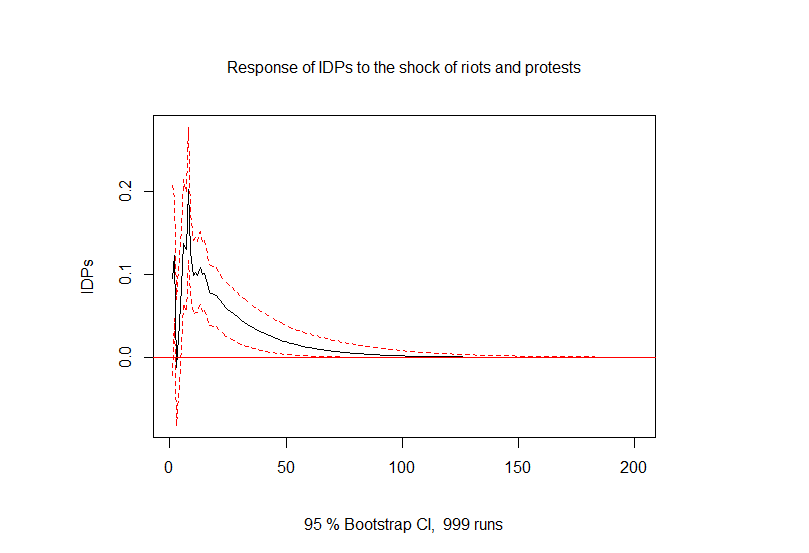

Supplement: S35 Fig — The highest positive effect of riots and protests on the number of IDPs is observed in the eighth week. Sources: ACLED and Humanitarian Tools database. (TIF) [file pntd.0011597.s035.tif]

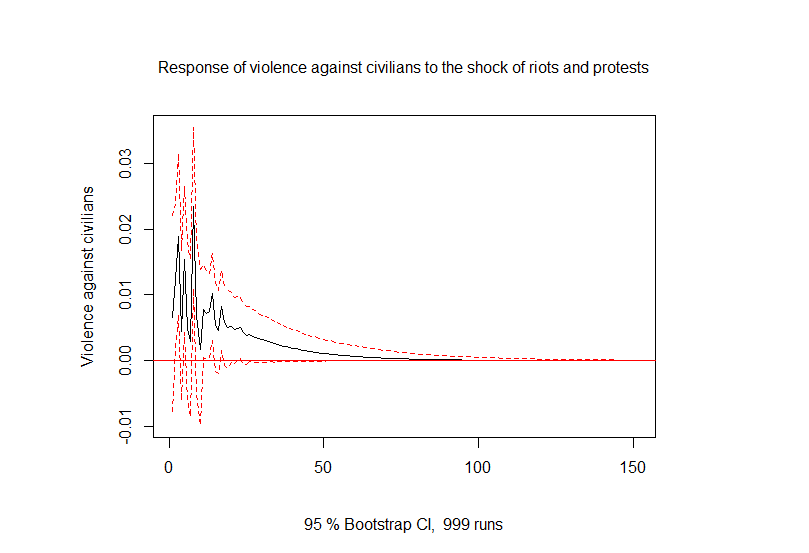

Supplement: S36 Fig — The highest positive effect of riots and protests on violence against civilians is observed in the eighth week. Source: ACLED. (TIF) [file pntd.0011597.s036.tif]

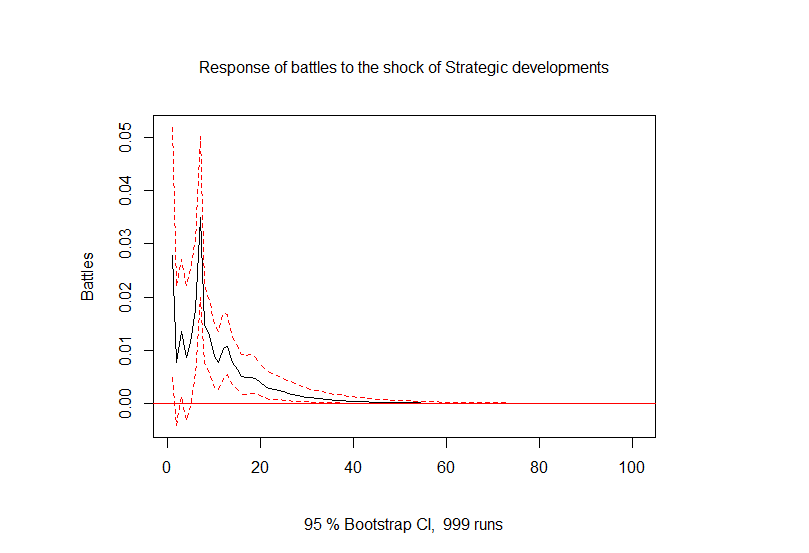

Supplement: S37 Fig — The highest positive effect of strategic developments on battles is observed in the first week. Source: ACLED. (TIF) [file pntd.0011597.s037.tif]

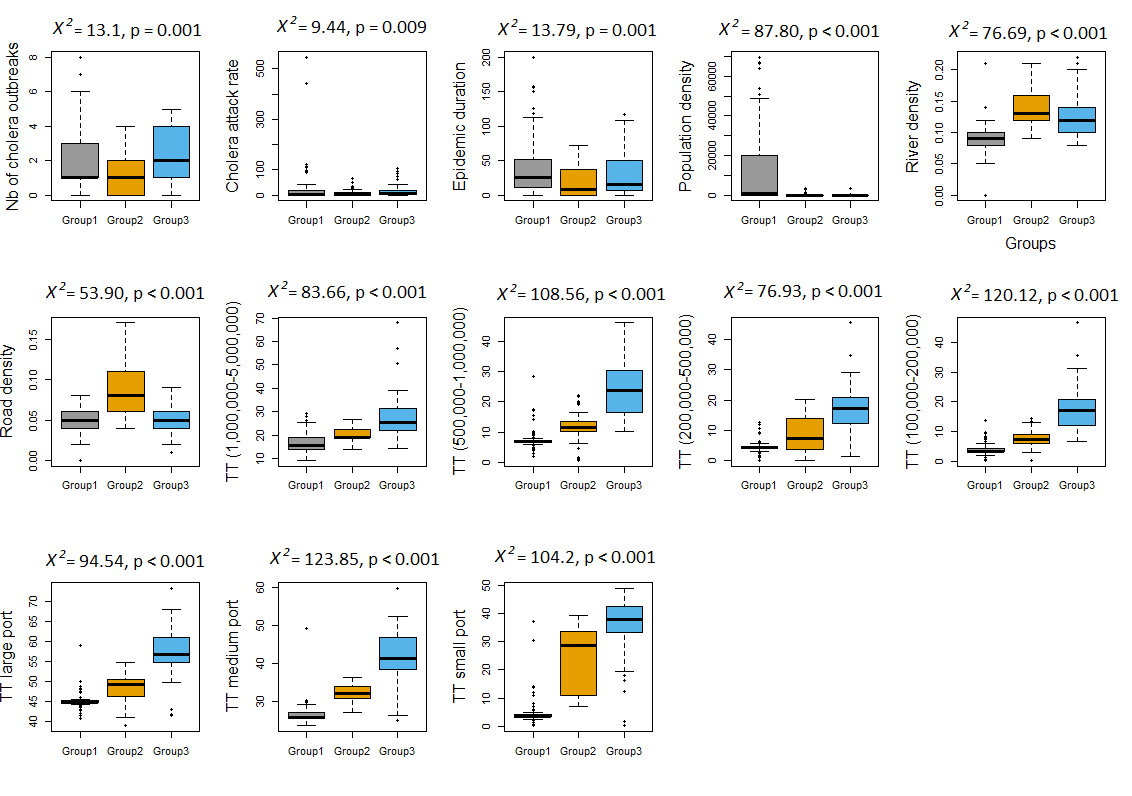

Supplement: S38 Fig — Sources: DRC’s IDSRS, WorldPop, Humanitarian Data Exchange, and Global travel-time accessibility indicators. (TIF) [file pntd.0011597.s038.tif]

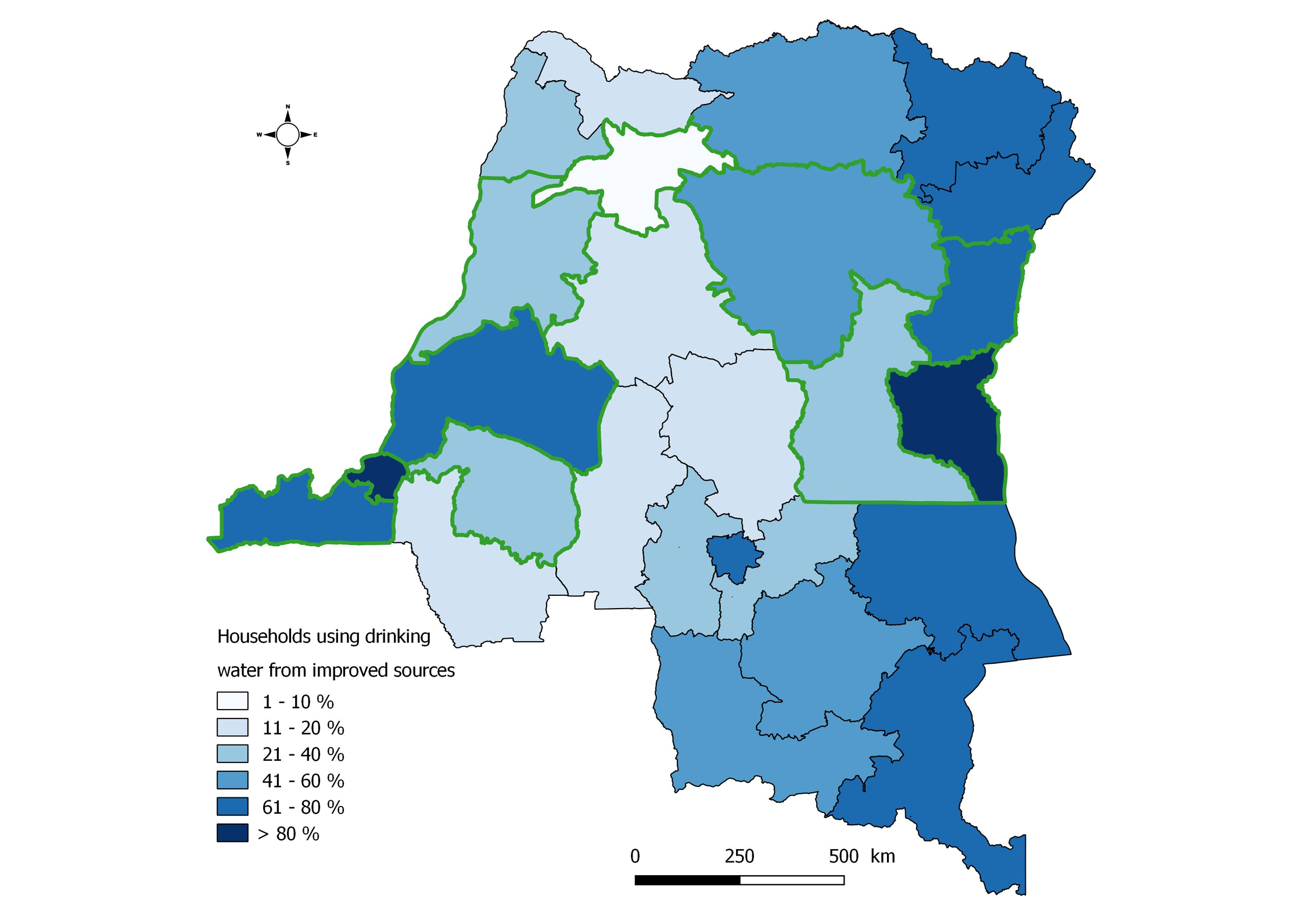

Supplement: S39 Fig — The green borders correspond to provinces involved in our study. Map produced in Quantum GIS version 3.8.3. using free open shapefiles of the first level administrative boundaries of the DRC from https://data.humdata.org/dataset/wfp-geonode-drc-first-level-administrative-boundaries. Source: Multiple Indicator Cluster Survey. (TIF) [file pntd.0011597.s039.tif]

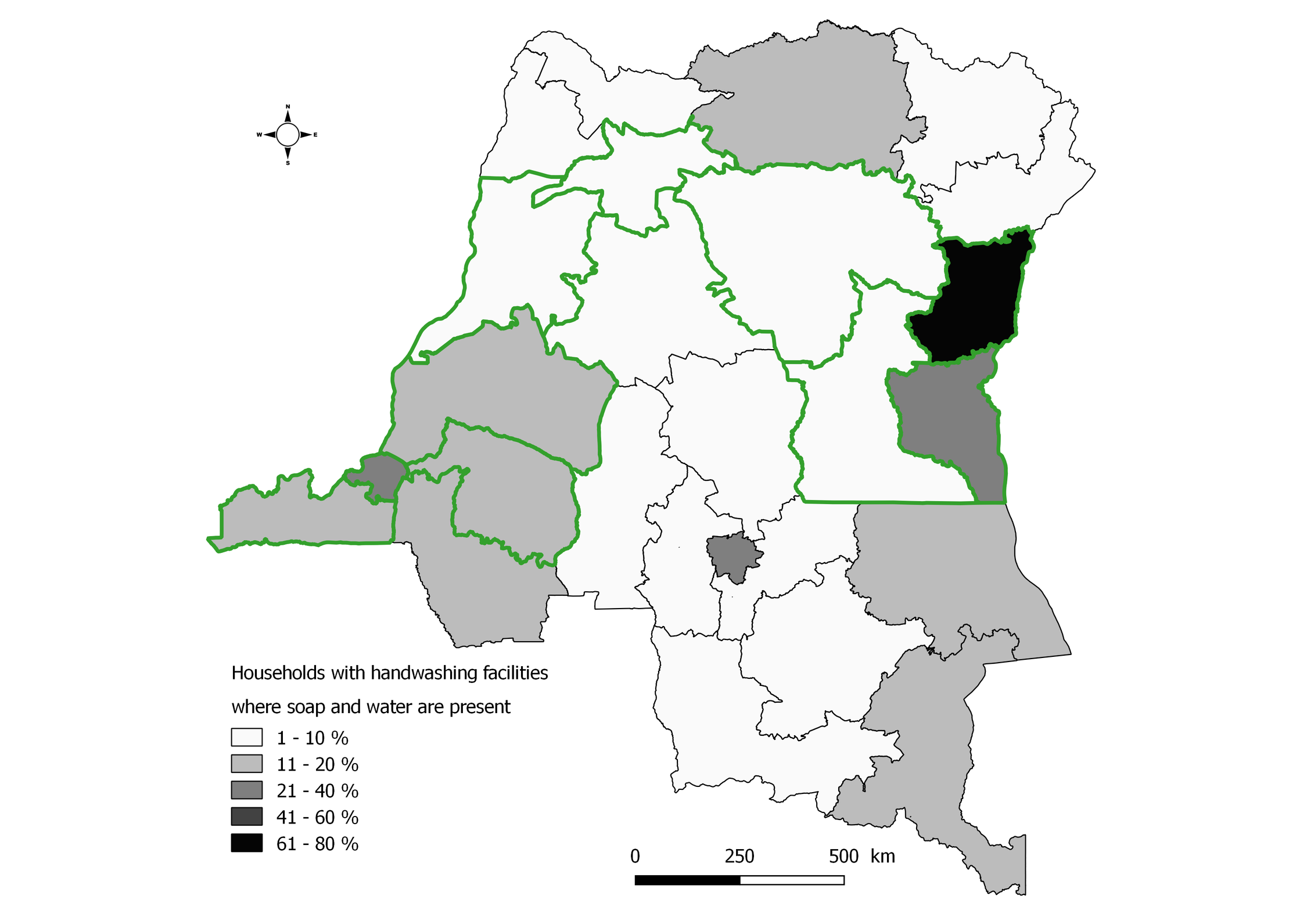

Supplement: S40 Fig — The green borders correspond to provinces involved in our study. Map produced in Quantum GIS version 3.8.3. using free open shapefiles of the first level administrative boundaries of the DRC from https://data.humdata.org/dataset/wfp-geonode-drc-first-level-administrative-boundaries. Source: Multiple Indicator Cluster Survey. (TIF) [file pntd.0011597.s040.tif]

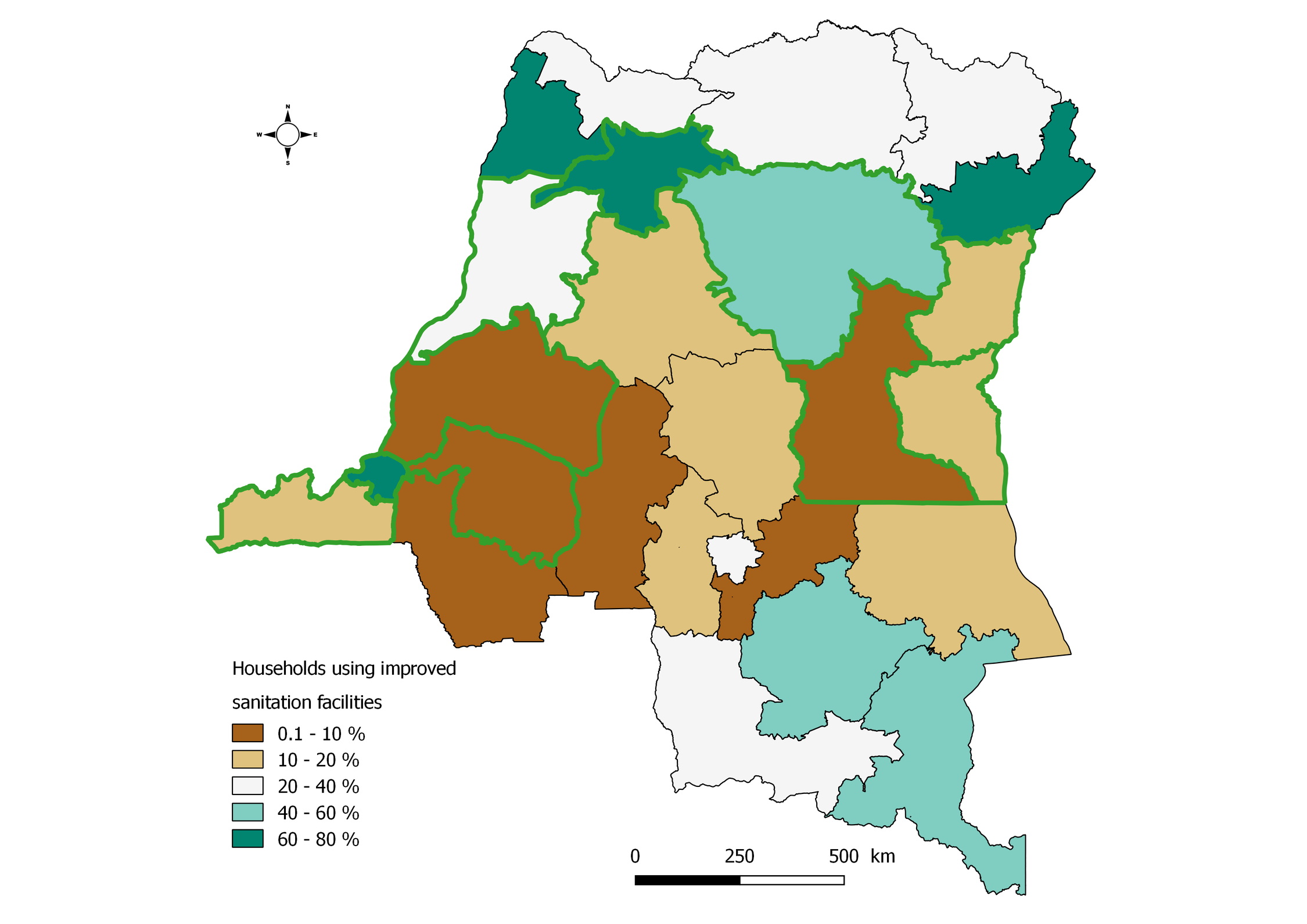

Supplement: S41 Fig — The green borders correspond to provinces involved in our study. Map produced in Quantum GIS version 3.8.3. using free open shapefiles of the first level administrative boundaries of the DRC from https://data.humdata.org/dataset/wfp-geonode-drc-first-level-administrative-boundaries. Source: Multiple Indicator Cluster Survey. (TIF) [file pntd.0011597.s041.tif]

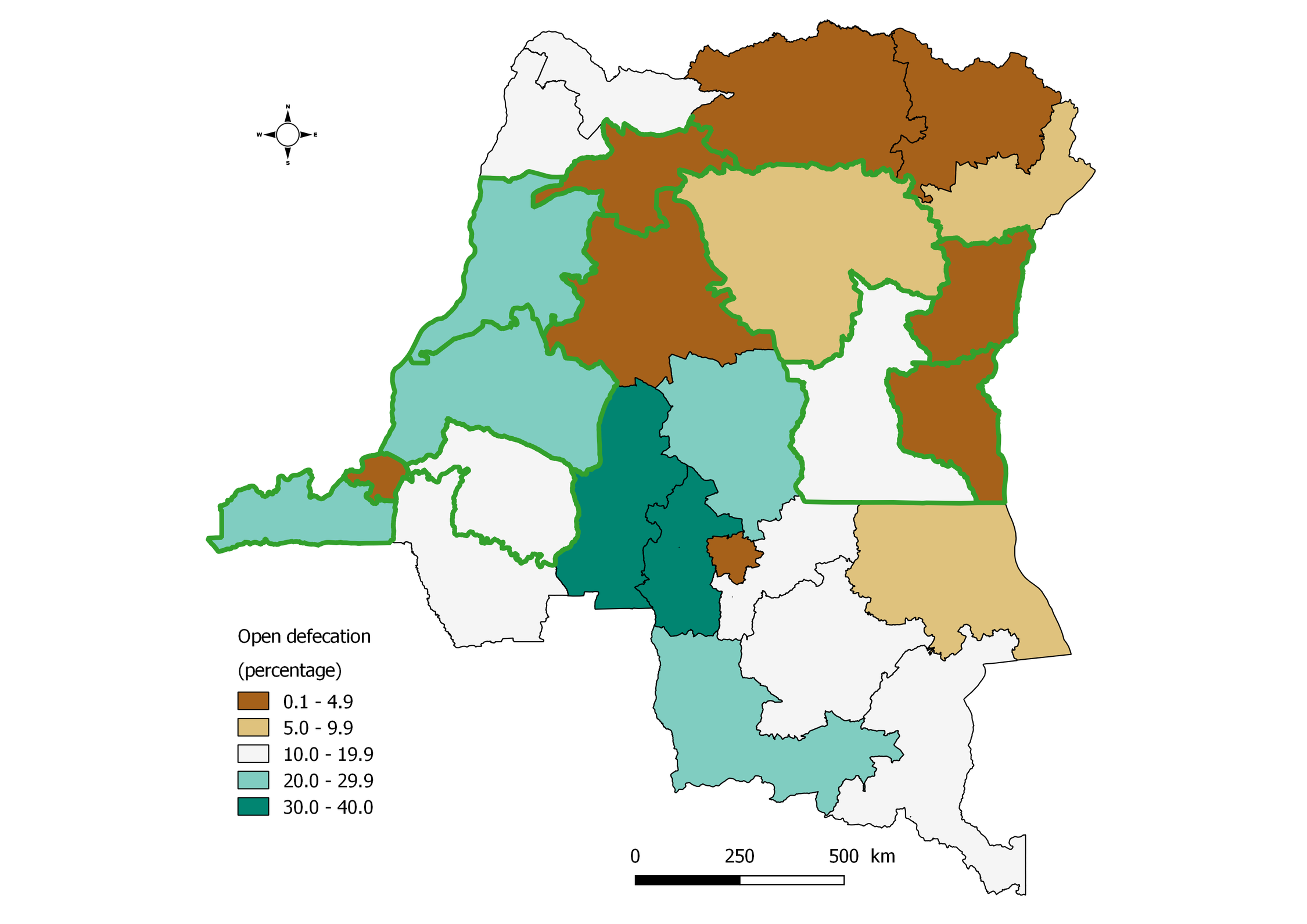

Supplement: S42 Fig — The green borders correspond to provinces involved in our study. Map produced in Quantum GIS version 3.8.3. using free open shapefiles of the first level administrative boundaries of the DRC from https://data.humdata.org/dataset/wfp-geonode-drc-first-level-administrative-boundaries. Source: Multiple Indicator Cluster Survey. (TIF) [file pntd.0011597.s042.tif]
