# Supplementary material for: Drivers of the dynamics of the spread of cholera in the Democratic Republic of the Congo, 2000–2018: An eco-epidemiological study
Source: PLoS Negl Trop Dis. 2023 Aug 28;17(8):e0011597. doi: 10.1371/journal.pntd.0011597 (PMC10491302; doi:10.1371/journal.pntd.0011597)
Supplement: S1 Table — Source: DRC’s IDSRS. (DOCX) [file pntd.0011597.s043.docx]

**Distribution of cholera**

**S1 Table. Annual number of suspected cholera cases reported in the DRC and Kivu provinces, 2000-2018**

| **Years** | **DRC** | **North Kivu** | **South Kivu** | **Endemic areas bordering Lake Kivu** |
| --- | --- | --- | --- | --- |
| 2000 | 14,995 | 7,462 | 5,309 | 5,915 |
| 2001 | 5,728 | 3,015 | 2,799 | 3,027 |
| 2002 | 31,658 | 3,426 | 4,183 | 2,305 |
| 2003 | 27,272 | 3,090 | 3,792 | 3,095 |
| 2004 | 7,665 | 2,855 | 3,340 | 1,241 |
| 2005 | 13,430 | 6,032 | 4,019 | 5,259 |
| 2006 | 20,642 | 5,533 | 11,715 | 1,0344 |
| 2007 | 28,269 | 6,787 | 11,562 | 8,420 |
| 2008 | 30,150 | 11,042 | 7,211 | 7,114 |
| 2009 | 22,899 | 6,233 | 10,950 | 7,511 |
| 2010 | 13,884 | 4,750 | 9,359 | 4,913 |
| 2011 | 21,700 | 5,640 | 6,590 | 3,741 |
| 2012 | 33,661 | 8,590 | 4,808 | 6,440 |
| 2013 | 26,944 | 4,449 | 7,294 | 5,392 |
| 2014 | 22,203 | 4,028 | 7,775 | 2,823 |
| 2015 | 19,182 | 2,883 | 4,387 | 2,435 |
| 2016 | 28,093 | 3,526 | 5,095 | 1,780 |
| 2017 | 56,190 | 14,280 | 11,987 | 16,732 |
| 2018 | 30,768 | 2,628 | 4,737 | 1,670 |
