# Supplementary material for: Drivers of the dynamics of the spread of cholera in the Democratic Republic of the Congo, 2000–2018: An eco-epidemiological study
Source: PLoS Negl Trop Dis. 2023 Aug 28;17(8):e0011597. doi: 10.1371/journal.pntd.0011597 (PMC10491302; doi:10.1371/journal.pntd.0011597)
Supplement: S2 Table — (DOCX) [file pntd.0011597.s044.docx]

**Distribution of types of conflicts**

**S2 Table. Summary of conflict events reported in the DRC, 2000-2018**

| **Conflict event** | **n** | **%** |
| --- | --- | --- |
| Battles | 5,337 | 44.5 |
| Remote violence | 111 | 0.9 |
| Riots and protests | 1,423 | 11.9 |
| Strategic developments | 1,034 | 8.7 |
| Violence against civilians | 4,075 | 34.3 |
| Total | 11,980 | 100 |
