# Supplementary material for: Drivers of the dynamics of the spread of cholera in the Democratic Republic of the Congo, 2000–2018: An eco-epidemiological study
Source: PLoS Negl Trop Dis. 2023 Aug 28;17(8):e0011597. doi: 10.1371/journal.pntd.0011597 (PMC10491302; doi:10.1371/journal.pntd.0011597)
Supplement: S3 Table — Source: ACLED. (DOCX) [file pntd.0011597.s045.docx]

**Distribution of types of conflicts at the province level**

**S3 Table. Summary of types of conflict events reported by provinces, 2000-2018**

| **Province** | **Battle**  **n (%)** | **Strategic developments**  **n (%)** | **Riots and protests**  **n (%)** | **Violence against civilians**  **n (%)** | **Remote violence**  **n (%)** |
| --- | --- | --- | --- | --- | --- |
| Bas Uele | 33 (0.6) | 12 (1.2) | 26 (1.8) | 144 (3.5) | 0 (0.0) |
| Equateur | 30 (0.6) | 5 (0.5) | 40 (2.8) | 19 (0.5) | 1 (0.9) |
| Haut Katanga | 164 (3.1) | 35 (3.4) | 114 (8.0) | 158 (3.9) | 5 (4.5) |
| Haut Lomami | 33 (0.6) | 4 (0.4) | 18 (1.3) | 27 (0.7) | 1 (0.9) |
| Haut Uele | 255 (4.8) | 75 (7.3) | 21 (1.5) | 468 (11.5) | 0 (0.0) |
| Ituri | 1,048 (19.6) | 71 (6.9) | 112 (7.9) | 491 (12.0) | 7 (6.3) |
| Kasaï | 26 (0.5) | 7 (0.7) | 12 (0.8) | 44 (1.1) | 2 (1.8) |
| Kasaï Central | 69 (1.3) | 20 (1.9) | 24 (1.7) | 67 (1.6) | 0 (0.0) |
| Kasaï Oriental | 16 (0.3) | 5 (0.5) | 60 (4.2) | 28 (0.7) | 0 (0.0) |
| Kinshasa | 43 (0.8) | 66 (6.4) | 226 (15.9) | 88 (2.2) | 3 (2.7) |
| Kongo Central | 23 (0.4) | 5 (0.5) | 29 (2.0) | 25 (0.6) | 0 (0.0) |
| Kwango | 3 (0.1) | 1 (0.1) | 0 (0.0) | 10 (0.2) | 0 (0.0) |
| Kwilu | 11 (0.2) | 6 (0.6) | 35 (2.5) | 13 (0.3) | 0 (0.0) |
| Lomami | 32 (0.6) | 1 (0.1) | 9 (0.6) | 14 (0.3) | 2 (1.8) |
| Lualaba | 12 (0.2) | 3 (0.3) | 17 (1.2) | 11 (0.3) | 0 (0.0) |
| Maï Ndombe | 5 (0.1) | 1 (0.1) | 5 (0.4) | 6 (0.1) | 2 (1.8) |
| Maniema | 121 (2.3) | 19 (1.8) | 25 (1.8) | 58 (1.4) | 1 (0.9) |
| Mongala | 16 (0.3) | 0 (0.0) | 3 (0.2) | 10 (0.2) | 0 (0.0) |
| North Kivu | 1,887 (35.4) | 412 (39.8) | 331 (23.3) | 1,411 (34.6) | 51 (45.9) |
| North Ubangi | 6 (0.1) | 2 (0.2) | 12 (0.8) | 13 (0.3) | 0 (0.0) |
| Sankuru | 17 (0.3) | 3 (0.3) | 10 (0.7) | 19 (0.5) | 0 (0.0) |
| South Kivu | 971 (18.2) | 186 (18.0) | 179 (12.6) | 714 (17.5) | 15 (13.5) |
| South Ubangi | 47 (0.9) | 9 (0.9) | 11 (0.8) | 34 (0.8) | 11 (9.9) |
| Tanganyika | 164 (3.1) | 54 (5.2) | 31 (2.2) | 121 (3.0) | 4 (3.6) |
| Tshopo | 67 (1.3) | 30 (2.9) | 68 (4.8) | 76 (1.9) | 2 (1.8) |
| Tshuapa | 238 (4.5) | 2 (0.2) | 5 (0.4) | 6 (0.1) | 4 (3.6) |
