# Supplementary material for: Drivers of the dynamics of the spread of cholera in the Democratic Republic of the Congo, 2000–2018: An eco-epidemiological study
Source: PLoS Negl Trop Dis. 2023 Aug 28;17(8):e0011597. doi: 10.1371/journal.pntd.0011597 (PMC10491302; doi:10.1371/journal.pntd.0011597)
Supplement: S4 Table — Source: ACLED. (DOCX) [file pntd.0011597.s046.docx]

**Distribution of types of conflicts in the Kivu provinces, and areas bordering Lake Kivu**

As observed at the national scale, battles were in the majority (n = 2,858), followed by violence against in the Kivu provinces (n = 2,125). In contrast, strategic developments (n = 598) were more recorded than riots and protests (n = 510). Furthermore, battles (n = 908) were the most reported conflict events in areas bordering Lake Kivu, followed by violence against civilians (n = 709), riots and protests (n = 260), and strategic developments (n = 226).

**S4 Table. Summary of types of conflict events reported in the Kivu provinces, and areas bordering Lake Kivu, 2000-2018**

| **Conflict event** | **Kivu provinces**  **N** | **Lake Kivu areas**  **n (%)** |
| --- | --- | --- |
| Battles | 2,858 | 908 (31.8) |
| Strategic developments | 598 | 226 (37.8) |
| Riots and protests | 510 | 260 (51.0) |
| Violence against civilians | 2,125 | 709 (33.4) |
