# Supplementary material for: Drivers of the dynamics of the spread of cholera in the Democratic Republic of the Congo, 2000–2018: An eco-epidemiological study
Source: PLoS Negl Trop Dis. 2023 Aug 28;17(8):e0011597. doi: 10.1371/journal.pntd.0011597 (PMC10491302; doi:10.1371/journal.pntd.0011597)
Supplement: S5 Table — Source: ACLED. (DOCX) [file pntd.0011597.s047.docx]

**Distribution of types of conflicts in areas around those bordering Lake Kivu according to cholera status**

For the areas surrounding those bordering Lake Kivu, each type of conflict event was recorded more in highly cholera-affected health zones than in less or unaffected health zones: battles (+81%), strategic developments (+ 76%), riots and protests (+ 71%), and violence against civilians (+ 59%).

**S5 Table. Summary of types of conflict events reported in areas around those bordering Lake Kivu according to cholera status, 2000-2018**

| **Conflict event** | **Kivu provinces**  **N** | **Areas heavily affected by cholera**  **n (%)** | **Areas less or unaffected by cholera**  **n (%)** |
| --- | --- | --- | --- |
| Battles | 2,858 | 630 (22.0) | 119 (4.2) |
| Strategic developments | 598 | 112 (18.7) | 27 (4.5) |
| Riots and protests | 510 | 28 (5.5) | 8 (1.6) |
| Violence against civilians | 2,125 | 308 (14.5) | 126 (5.9) |
