# Supplementary material for: Drivers of the dynamics of the spread of cholera in the Democratic Republic of the Congo, 2000–2018: An eco-epidemiological study
Source: PLoS Negl Trop Dis. 2023 Aug 28;17(8):e0011597. doi: 10.1371/journal.pntd.0011597 (PMC10491302; doi:10.1371/journal.pntd.0011597)
Supplement: S6 Table — Source: Humanitarian Tools database. (DOCX) [file pntd.0011597.s048.docx]

**Distribution of the number of IDPs at the province level**

**S6 Table. Summary of the number of IDPs reported by provinces, 2009-2018**

| **Provinces** | **n** | **%** | **Reporting periods** |
| --- | --- | --- | --- |
| Bas Uele | 0 | 0 | - |
| Equateur | 0 | 0 | - |
| Haut Katanga | 119,314 | 2 | 2013-2018 |
| Haut Lomami | 45,291 | 0.7 | 2016-2018 |
| Haut Uele | 995 | 0 | - |
| Ituri | 886,238 | 15.3 | 2016-2018 |
| Kasaï | 359,801 | 6.2 | 2016-2018 |
| Kasaï Central | 569,421 | 9.8 | 2016-2018 |
| Kasaï Oriental | 77,964 | 1.3 | 2016-2018 |
| Kinshasa | 0 | 0 | - |
| Kongo Central | 0 | 0 | - |
| Kwango | 1,815 | 0 | 2017 |
| Kwilu | 30,050 | 0.5 | 2017 |
| Lomami | 246,810 | 4.2 | 2016-2018 |
| Lualaba | 9,210 | 0.1 | 2013-2018 |
| Maï Ndombe | 0 | 0 | - |
| Maniema | 348,626 | 6 | 2011-2018 |
| Mongala | 0 | 0 | - |
| North Kivu | 1,757,018 | 30.4 | 2009-2018 |
| North Ubangi | 0 | 0 | - |
| Sankuru | 0 | 0 | - |
| South Kivu | 842,789 | 14.5 | 2009-2018 |
| South Ubangi | 0 | 0 | - |
| Tanganyika | 429,921 | 7.4 | 2014-2018 |
| Tshopo | 51,311 | 0.8 | - |
| Tshuapa | 0 | 0 | - |
| Total | 5,776,574 | 100.0 |  |
