# Supplementary material for: Drivers of the dynamics of the spread of cholera in the Democratic Republic of the Congo, 2000–2018: An eco-epidemiological study
Source: PLoS Negl Trop Dis. 2023 Aug 28;17(8):e0011597. doi: 10.1371/journal.pntd.0011597 (PMC10491302; doi:10.1371/journal.pntd.0011597)
Supplement: S7 Table — Source: Humanitarian Tools database. (DOCX) [file pntd.0011597.s049.docx]

**Distribution of the number of IDPs in the Kivu provinces, and cholera endemic areas bordering Lake Kivu**

**S7 Table. Summary of the number of IDPs reported in the Kivu provinces, and cholera endemic areas bordering Lake Kivu, 2009-2018**

| **Years** | **Kivu provinces**  **N** | **Lake Kivu areas**  **n (%)** |
| --- | --- | --- |
| 2009 | 14,036 | 0 (0.0) |
| 2010 | 14,322 | 950 (6.6) |
| 2011 | 13,740 | 0 (0.0) |
| 2012 | 97,243 | 18,915 (19.5) |
| 2013 | 59,207 | 0 (0.0) |
| 2014 | 40,002 | 280 (0.7) |
| 2015 | 107,808 | 14,735 (13.7) |
| 2016 | 582,260 | 8,102 (1.4) |
| 2017 | 924,369 | 19,559 (2.1) |
| 2018 | 746,820 | 10,837 (1.5) |
| Total | 2,599,807 | 76,880 (3.0) |
