# Supplementary material for: Drivers of the dynamics of the spread of cholera in the Democratic Republic of the Congo, 2000–2018: An eco-epidemiological study
Source: PLoS Negl Trop Dis. 2023 Aug 28;17(8):e0011597. doi: 10.1371/journal.pntd.0011597 (PMC10491302; doi:10.1371/journal.pntd.0011597)
Supplement: S8 Table — Source: Humanitarian Tools database. (DOCX) [file pntd.0011597.s050.docx]

**Distribution of the number of IDPs in areas around those bordering Lake Kivu according to cholera status**

**S8 Table. Summary of the number of IDPs reported in areas around those bordering Lake Kivu according to cholera status, 2009-2018**

| **Years** | **Kivu provinces**  **N** | **Areas heavily affected by cholera**  **n (%)** | **Areas less or unaffected by cholera**  **n (%)** |
| --- | --- | --- | --- |
| 2009 | 14,036 | 6,553 (46.7) | 0 (0.0) |
| 2010 | 14,322 | 0 (0.0) | 1,190 (8.3) |
| 2011 | 13,740 | 0 (0.0) | 1,655 (12.0) |
| 2012 | 97,243 | 5,398 (5.6) | 50,205 (51.6) |
| 2013 | 59,207 | 1,572 (2.7) | 0 (0.0) |
| 2014 | 40,002 | 880 (2.2) | 0 (0.0) |
| 2015 | 107,808 | 0 (0.0) | 0 (0.0) |
| 2016 | 582,260 | 96,918 (16.6) | 4,406 (0.8) |
| 2017 | 924,369 | 239,034 (25.9) | 26,018 (2.8) |
| 2018 | 746,820 | 69,957 (9.4) | 20,768 (2.8) |
| Total | 2,599,807 | 420,312 (16.2) | 104,242 (4.0) |
